# Exosome-mediated delivery of an anti-angiogenic peptide inhibits pathological retinal angiogenesis

**DOI:** 10.7150/thno.54755

**Published:** 2021-03-05

**Authors:** Xue Dong, Yi Lei, Zeyang Yu, Tian Wang, Yi Liu, Gang Han, Xiaodan Zhang, Yiming Li, Yinting Song, Heping Xu, Mei Du, Haifang Yin, Xiaohong Wang, Hua Yan

**Affiliations:** 1 Department of Ophthalmology, Tianjin Medical University General Hospital, 300070, Tianjin, China.; 2 Department of Pharmacology and Tianjin Key Laboratory of Inflammation Biology, The Province and Ministry Co-sponsored Collaborative Innovation Center for Medical Epigenetics, School of Basic Medical Sciences, Tianjin Medical University, 300070, Tianjin, China.; 3 Laboratory of Molecular Ophthalmology, Tianjin Medical University.; 4 School of Medical Laboratory, Tianjin Medical University, Guangdong Road, Tianjin 300203, China.; 5 The Wellcome-Wolfson Institute for Experimental Medicine, Queen's University Belfast, 97 Lisburn Road, Belfast, BT9 7BL, UK.; 6 Tianjin Key Laboratory of Cellular Homeostasis and Human Diseases & Department of Cell Biology, Tianjin Medical University, Qixiangtai Road, Heping District, Tianjin, 300070, China.

**Keywords:** Exosomes (EXOs), drug delivery, proliferative retinopathy, pathological angiogenesis, VEGF signaling.

## Abstract

**Background:** Pathological angiogenesis is the hallmark of many vision-threatening diseases. Anti-VEGF is a primary treatment with substantial beneficial effects. However, such agents require frequent intravitreal injections. Our previous work established a method for effectively modifying exosomes (EXOs) for loading therapeutic peptides. Here, we used this system to load the anti-angiogenic peptide KV11, aiming to establish an EXO-based therapy strategy to suppress neovascularization in the retina.

**Methods:** Using an anchoring peptide, CP05, we linked KV11 to endothelial cell (EC) derived EXOs, yielding EXO_KV11_. We tested the delivery efficiency of EXO_KV11_ via two commonly used ocular injection methods: retro-orbital injection and intravitreal injection. Deploying an oxygen-induced retinopathy (OIR) model and a VEGF injection model, we tested the effects of EXO_KV11_ on neovascular formation, EC proliferation, and vascular permeability. *In vitro* experiments were used to test the mechanism and to analyze the effects of EXO_KV11_ on EC proliferation, migration, and sprouting.

**Results**: By using the EXO loading system, KV11 was more efficiently delivered to the blood vessels of the mouse retina via retro-orbital injection. In both OIR model and VEGF injection model, EXO_KV11_ was more effective than KV11 alone in inhibiting neovascularization and vessel leakage. The therapeutic effect of retro-orbital injection of EXO_KV11_ was comparable to the intravitreal injection of VEGF-trap. Mechanistically, KV11 alone inhibited VEGF-downstream signaling, while EXO_KV11_ showed a stronger effect.

**Conclusions:** We used EXOs as a carrier for intraocular delivery of KV11. We showed that KV11 itself has an anti-angiogenic effect through retro-orbital injection, but that this effect was greatly enhanced when delivered with EXOs. Thus, this system has the potential to treat proliferative retinopathy via retro-orbital injection which is a less invasive manner compared with intravitreal injection.

## Introduction

Angiogenesis is vital for embryonic development 1, 2, and abnormal blood vessel growth contributes to many diseases, such as tumors and neovascular eye diseases 3, 4. Pathological retinal neovascularization is a key pathological change in diseases such as retinopathy of prematurity (ROP) and proliferative diabetic retinopathy (PDR), which are the major causes of vision loss in preterm infants and working-age adults 5-7. Abnormal blood vessel growth is also characterized by compromised blood-retinal barrier (BRB) function, which further leads to hemorrhage, retinal detachment, and eventually blindness 8.

An elevated intraocular level of vascular endothelial growth factor (VEGF) is considered as the major cause that drives abnormal vessel growth and BRB breakdown. This has led to the use of intravitreal injection of VEGF inhibitors (such as the Fab-Fragment ranibizumab and VEGF decoy proteins aflibercept), which have been proven to have substantial beneficial effects 9, 10. However, whether or not anti-VEGF is detrimental for the retina is controversially discussed. Although some studies have reported that anti-VEGF injection is safe 11, 12, others have had a concern that a major potential risk of this treatment is that during long-term therapy 13, intravitreal injections need to be repeated, which is invasive and may cause blinding sequelae such as endophthalmitis and retinal detachment 14, 15. Furthermore, due to the neurosupportive properties of VEGF, some studies indicated that long-term of anti-VEGF treatment can also be associated with neuronal toxicity and developmental side effects 16-18. Thus, it remains desirable to develop safer alternative therapeutic methods.

Exosomes (EXOs) are nanoparticles with sizes of 50 to 150 nm that are secreted by many different cell types 19. EXOs are generated by the inward budding of endosomal membrane during maturation of multivesicular bodies (MVBs) and secreted into the extracellular environment upon fusion with the cell surface 20. Exosomes may carry a large variety of cargoes, including cytosolic and cell-surface proteins, lipids, nucleic acids and metabolites 21. Due to their natural, non-toxic, and biodegradable characteristics, EXOs are considered as ideal candidates for drug delivery to treat many diseases such as cancer, neurodegenerative disease, cardiovascular disease or muscular dystrophy 22, 23. EXOs carrying propeptide enhanced muscle regeneration and growth in muscular dystrophy 24. EXOs loaded with different cargoes, such as chemotherapeutics, multiple tumor-associated antigen or potent adjuvant, can enhance tumor recognition and suppress tumor growth 25, 26. Moreover, their ability to cross various biological barriers, including the BRB, highlights the potential of using EXOs as drug delivery carriers that can penetrate formidable biological barriers 27, 28. In the retina, EXO-associated AAV vectors have shown robust delivery efficiency into the murine retina compared with conventional AAV 29. Thus, we aimed to test whether EXOs could be loaded with therapeutics and introduced into the eyes for treating retinopathies. HUVECs (human umbilical vein endothelial cells), which are derived from the umbilical cord endothelium, have been used extensively for studying the function of endothelial cells (ECs). We chose HUVECs to produce endogenous EXOs, with the intention of increasing the potency of anti-angiogenesis peptide delivery into the retina vasculature.

CD63 is a tetraspanin that is enriched on the surface of EXOs. Using phage display, we previously identified a peptide, CP05, which bound specifically to CD63 and enabled direct painting and effective modification of EXOs 30. KV11, which consists of 11 amino acid residues from the human apolipoprotein(a) kringle 5-like domain (KV), showed a potential anti-angiogenic effect *in vitro* and *in vivo* 31, 32. In this study, we hypothesized that by using CP05, KV11 could be loaded on EXOs and efficiently delivered to ECs, thereby improving its therapeutic efficacy. We demonstrate that this system has the potential to treat proliferative retinopathy in a highly effective but less invasive way.

## Results

### EXOs mediate efficient cellular uptake of KV11 *in vitro*

To test the feasibility of using the CP05-EXO system as a delivery tool in ECs, we first isolated HUVEC-derived EXOs by centrifugation and examined their properties. Transmission electron microscopy image showed that EXOs had a typical sauce-cup shape (**Figure S1A**). Nanosight showed a peak diameter of 112.0 nm, which is within the normal size range 23 (**Figure S1B**). By labeling EXOs with DiR, we observed that EXOs can be effectively taken up by HUVECs (**Figure S1C**). Western blotting showed that isolated EXOs possessed a high level of EXO-associated markers compared with whole cell lysate of HUVECs or supernatant, including Alix and CD81. CD63 is particularly abundant in the isolated EXOs, which should allow further modification using the CP05 peptide system (**Figure S1D**).

**Figure 1:**
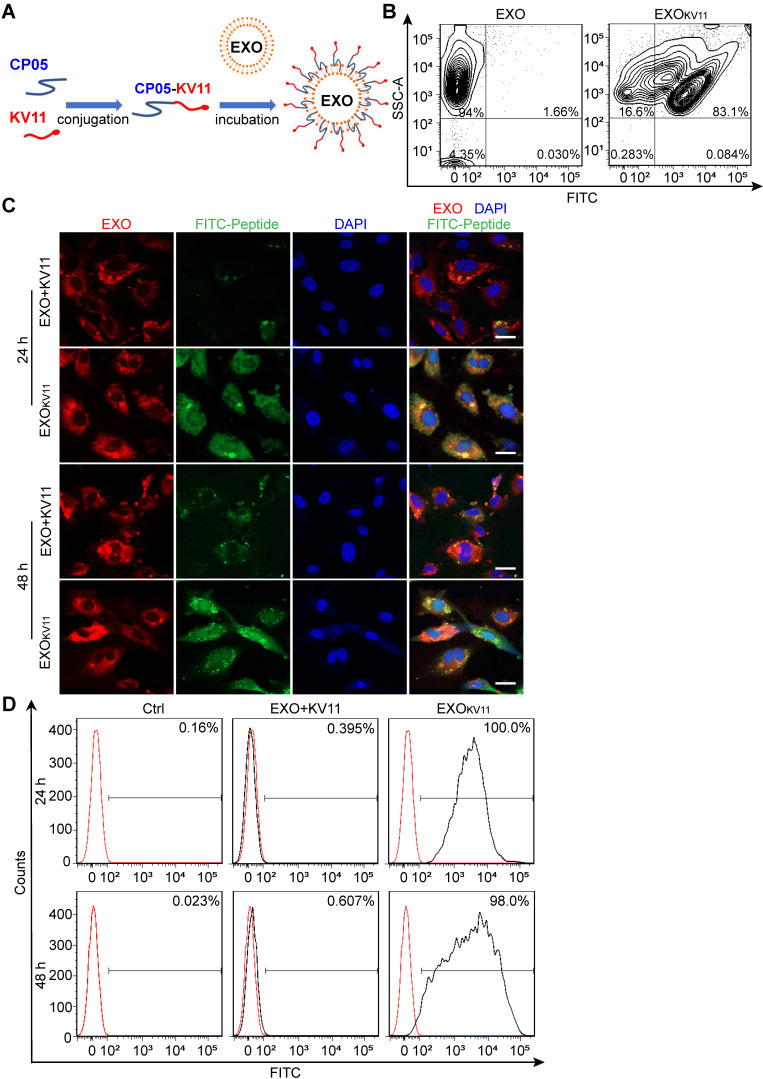
** Preparation of EXOs loaded with anti-angiogenesis peptide and efficiency of HUVEC delivery.** (A) Schematic illustration of preparation of EXO_KV11_ by painting an anti-angiogenic peptide (KV11) on EXOs via the exosomal anchor peptide CP05. (B) Flow cytometry for measuring the binding efficiency of KV11-CP05 on EXOs. For checking cellular uptake, FITC-labeled KV11-CP05 was used. (C) EXOs were labeled with DiR and incubated with FITC- labeled KV11 (EXO+KV11) or FITC- labeled KV11-CP05 (EXO_KV11_) for 6 h at 4 °C. Each mixture was then added to HUVECs in culture. Representative confocal images from at least three different experiments show the cellular uptake of KV11 in HUVECs at 24 and 48 h. (D) EXO+KV11 and EXO_KV11_ prepared as in (C) were added to HUVECs in culture. After 24 or 48 h, cells were trypsinized for flow cytometry to analyze the cellular uptake efficiency of EXO+KV11 and EXO_KV11_. The red line represents the no-treatment control (Ctrl). The percentage of FITC-positive events out of total events is indicated in each histogram. Scale bars, 20 µm in (C).

Next, we aimed to construct the EXO-linked KV11 peptide (EXO_KV11_) using CP05 as an anchor peptide (**Figure 1A**). For this, KV11-CP05 was incubated with EXOs for 6 h, followed by diafiltration to remove unbound peptides. For experiments intended to visualize EXO_KV11_, fluorescein isothiocyanate (FITC)-labeled KV11-CP05 was used. To evaluate whether CP05 can efficiently load KV11 onto EXOs, we performed flow cytometry for EXO_KV11_. The results showed that 83.1% of KV11 peptides were anchored on EXOs, indicating an efficient EXO loading system using CP05 (**Figure 1B**).

We then tested the delivery efficiency of EXO_KV11_ compared with a mixture of KV11 and EXOs in the absence of CP05 (EXO+KV11). For this, DiR-labeled EXOs were used to visualize EXO uptake; FITC-KV11-CP05 or FITC-KV11 were then incubated individually with DiR-labeled EXOs (**Figure 1C**). After incubation, unbound peptides were removed by diafiltration, and the products were then incubated with HUVECs or human retinal microvascular endothelial cells (HRMECs) for 24 or 48 h to test the delivery efficiency. Unsurprisingly, DiR-labeled EXOs could be taken up by both HUVECs and HRMECs (**Figure 1C, Figure S2A**). However, while strong FITC signals were observed in EXO_KV11_ (FITC-KV11-CP05 anchored EXOs), only very weak fluorescence signals were observed in EXO+KV11 (FITC-KV11 incubated with EXOs in the absence of CP05) group (**Figure 1C, Figure S2A**). Consistent with this, flow cytometry analysis also revealed that at 24 h,100% of HUVECs and HRMECs were FITC-positive in EXO_KV11_-treated group, while in the EXO+KV11 group the proportion of FITC^+^ cells was only 0.395% and 29.1%, respectively (**Figure 1D, Figure S2B**). Additionally, there were still 98.0% FITC^+^ HUVECs and 100% FITC^+^ HRMECs at 48 h, indicating that this system was stable (**Figure 1D, Figure S2B**). The above results strongly suggested EXO_KV11_ as an efficient delivery system *in vitro*.

### EXOs efficiently deliver an anti-angiogenic peptide into the retina vasculature

We then asked whether the above system could be used as a tool to deliver cargo into the retina vasculature *in vivo*. Intravitreal injection and retro-orbital injection are two commonly used ocular drug delivery methods, we tested the delivery efficiency of these two methods. Between them, intravitreal injection is known to be associated with the risk of retinal detachment, retinal hemorrhage and endophthalmitis 14, 15, while retro-orbital injection is a less invasive method with lower risks associated with the intravitreal route of administration 33-35.

**Figure 2:**
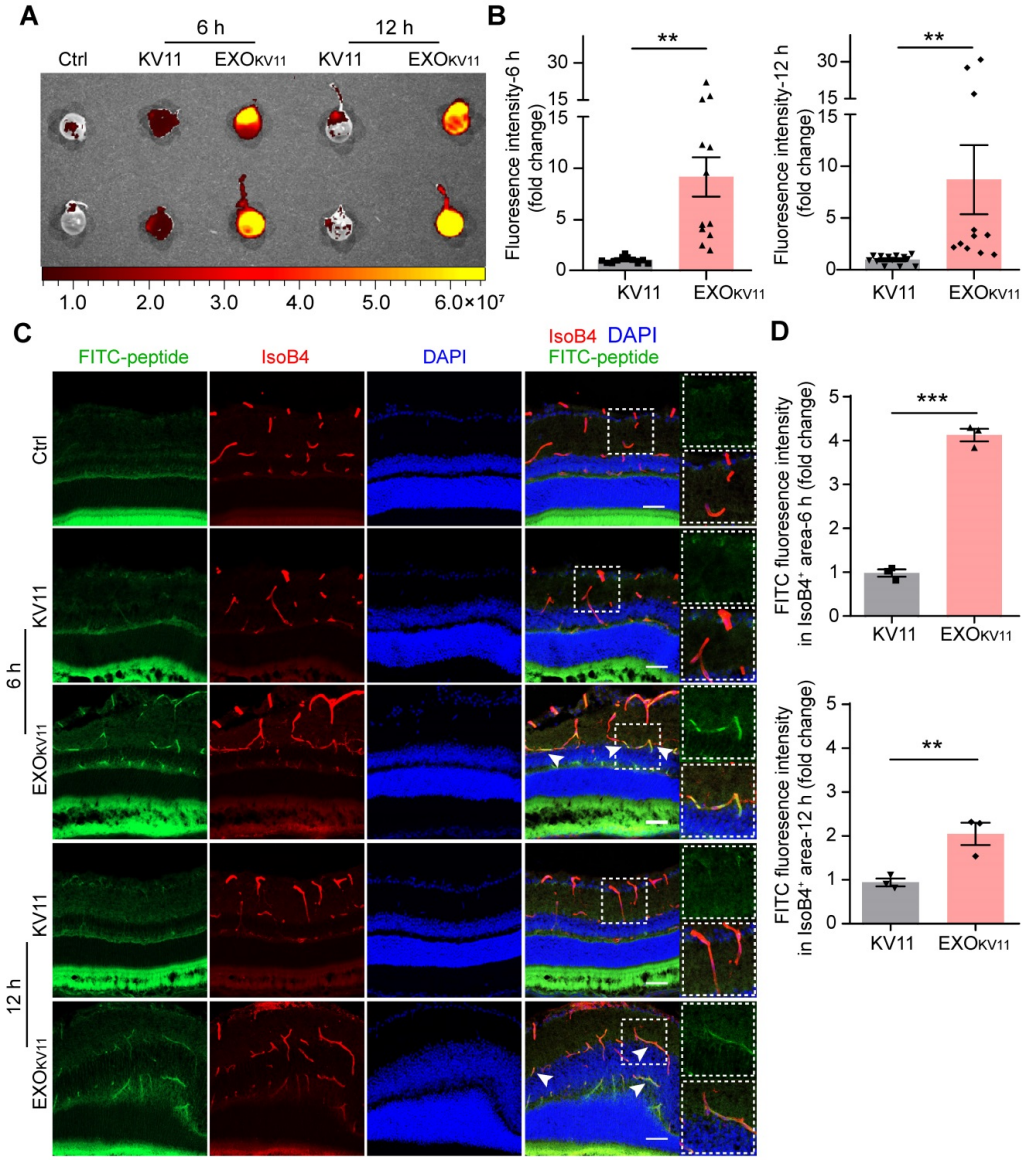
** Ocular delivery efficiency of EXO_KV11_.** (A) FITC-labeled KV11 or EXO_KV11_ was injected into CD-1 mice retro-orbitally. Fluorescence of the eyes was imaged at 6 and 12 h after the indicated injection. Ctrl represents the uninjected group. (B) Quantification of fluorescence intensity of eyes in (A) (n = 11 - 12 mice/group). (C) Ocular distribution of KV11 and EXO_KV11_ in cryosections of retinas from CD-1 mice treated as in (A). For checking *in vivo* distribution, FITC-labeled peptides were used. Arrowheads indicate co-localization of ECs (red, IsoB4) and peptide (green, FITC). (D) Quantification of fluorescence intensity of FITC-peptide in IsoB4^+^ area in cryosections of retinas in (C) (n = 3 mice/group; at least 3 images per mouse were analyzed and the values were averaged. The pictures for analysis were taken from the whole retinas, including peripheral part and retinas next to the optic nerve). The data represent as mean ± SEM. *p < 0.05, **p < 0.01, ***p < 0.001, Mann-Whitney test in (B), student's t test (two-tailed) in (D). Scale bars, 20 µm.

A previous study has also proved that retro-orbital injection method to be effective for perfusing retina vessels 36, thus, we first used retro-orbital injection to determine the delivery efficiency. We injected EXO_KV11_ and KV11 into CD-1 mice, an albino mouse strain that allows us to acquire images with an IVIS imaging system (**Figure 2A**). Six hours after injection, abundant FITC signals were detected in the eyes of mice injected with EXO_KV11_, while a much weaker signal was observed in KV11-injected group, suggesting that EXO_KV11_ was delivered into the eye more efficiently than KV11 (**Figure 2B**). Furthermore, at 12 h, the FITC signal was still detectable (**Figure 2B**), indicating high stability of EXO_KV11_. We then checked the distribution of EXO_KV11_ in the retina. For this, 6 h after retro-orbital injection, the eyes were dissected, snap-frozen and cryosectioned. Blood vessels were labeled with Isolectin B4 (IsoB4) (**Figure 2C**). Confocal images showed that the FITC signal was strongly enriched in blood vessels in EXO_KV11_-injected eyes, but much more weakly in KV11-injected ones (**Figure 2C,D**). Similarly, FITC signals were still detectable in the retina vasculature 12 h after injection, further highlighting the high stability of this system.

Intravitreal injection is another common method for delivering drugs into the eye. We next explored the delivery efficiency of EXO_KV11_ via intravitreal administration. To this end, we dissected eyes at 6 or 12 h after intravitreal injection of KV11 or EXO_KV11_. Confocal images showed that regardless of injection with either KV11 or EXO_KV11_, there was no clear co-localization of FITC signals and blood vessels (**Figure S3**).

Taken together, the above- mentioned results suggested that retro-orbital injection of a CP05-linked anti-angiogenic peptide and EXOs can be used as an efficient method for targeting retinal blood vessels, highlighting the possibility of treating pathological angiogenesis in the retina.

### EXO_KV11_ suppresses neovascularization of the retina in the OIR model

**Figure 3:**
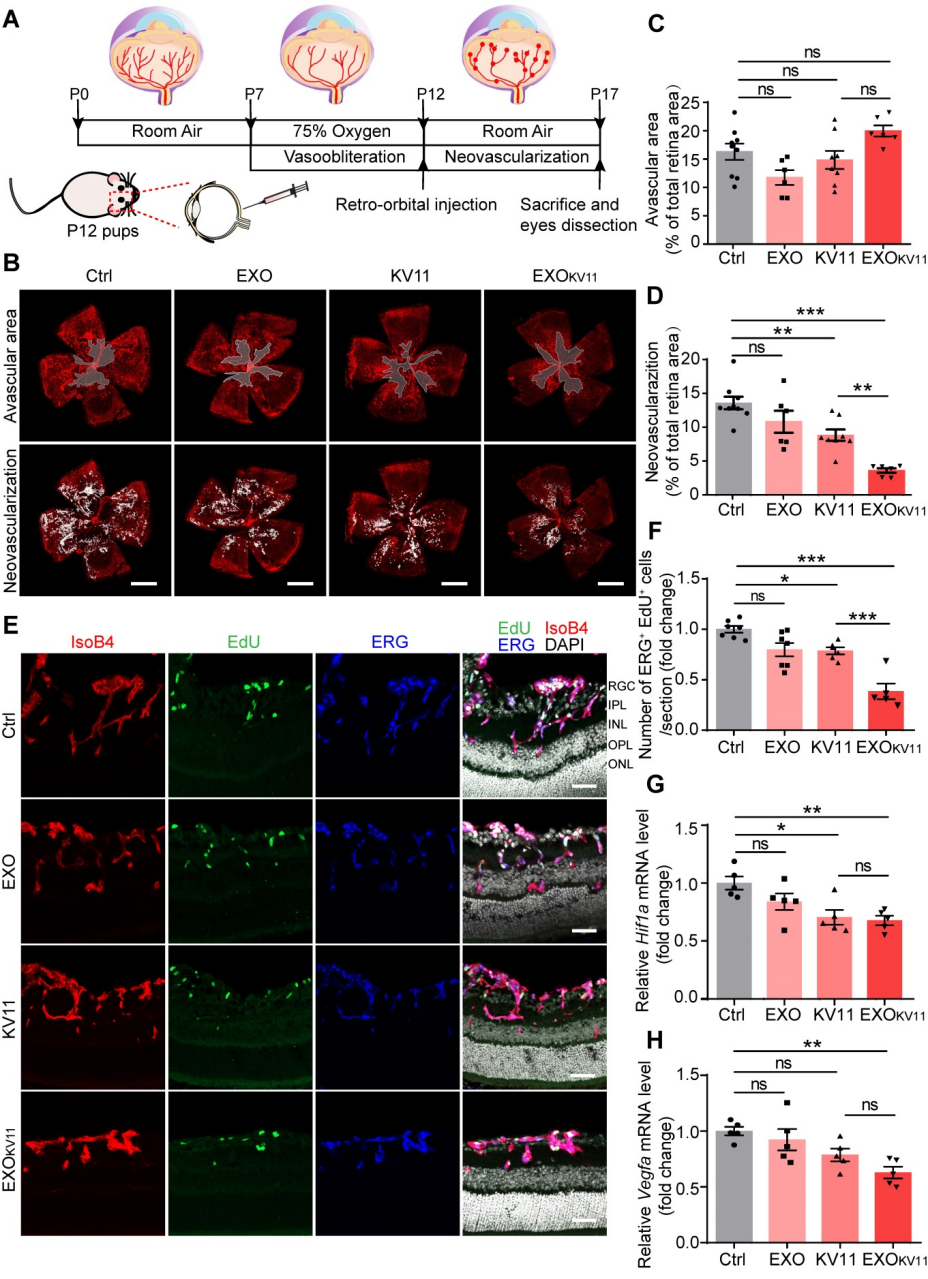
** EXO_KV11_ inhibits retinal neovascularization and suppresses EC proliferation in the OIR model.** (A) Schematic depiction of the mouse OIR model. Pups were placed in 75% oxygen from P7 to P12, and then returned to normal oxygen conditions. Saline vehicle (Ctrl), EXO, KV11, or EXO_KV11_ were injected retro-orbitally at P12. Pups were sacrificed and eyes were dissected at P17. (B) Representative confocal images of retina vasculature stained with IsoB4 in OIR retinas of saline vehicle (Ctrl)-, EXO-, KV11-, and EXO_KV11_-treated pups at P17. Upper panels indicate the avascular area and lower panels show the neovascular tufts. (C and D) Avascular area and neovascularization quantification of (B) (n = 6 - 9 eyes per condition). (E) Representative confocal images of retina sagittal sections costained with IsoB4, EdU (labels proliferating cells), and ERG (labels EC nuclei) in pups treated as in (A). (F) Quantitative analysis of EdU^+^ ERG^+^ cell number in (E) (n = 5 - 7 per condition). (G and H) qPCR analysis of the expression of *Hif1a* and *Vegfa* in OIR retinas of saline vehicle (Ctrl)-, EXO-, KV11-, and EXO_KV11_-treated pups at P17 (n = 5 per condition). The data represent as mean ± SEM, *p < 0.05, **p < 0.01, ***p < 0.001, one-way ANOVA followed by Tukey's multiple comparisons test in (C), (D), (F), (G), (H). Scale bars, 1 mm in (B), 50 µm in (E).

The above results encouraged us to test the therapeutic effects of EXO_KV11_ in the oxygen-induced retinopathy (OIR) mouse model, which resembles human ROP and certain aspects of human PDR 37-39. Mouse pups were exposed to hyperoxia conditions (75% oxygen) from postnatal day 7 (P7) to P12 as previously described 38 (**Figure 3A**). Hyperoxia causes oxygen toxicity and reduces the level of VEGF, which leads to vaso-obliteration. At P12, when the pups were returned to ambient air, the relatively hypoxic environment caused upregulation of VEGF, which then led to pathological neovascularization, consisting of overgrowth of immature vasculature and the consequent formation of extraretinal vascular tufts **(Figure 3A)** 40, 41. To test whether EXO_KV11_ has anti-angiogenic effects in the OIR model, EXO, KV11 or EXO_KV11_ was retro-orbitally injected into the pups at P12. Saline vehicle (Ctrl) injected in the same way served as the control. Analysis of the avascular areas of the retina revealed that EXO_KV11_ did not alter vessel regression (**Figure 3B,C**). However, quantifying vessel tuft formation indicated that retinal neovascularization was significantly reduced in the KV11 and EXO_KV11_ groups **(Figure 3B,D)** 42. In addition, when we compared KV11 and EXO_KV11_, the latter showed a stronger inhibitory effect on neovascularization in the OIR model **(Figure 3B,D)**.

As we observed reduced neovascularization in the KV11- and EXO_KV11_-treated OIR model, we next assessed whether EC proliferation was affected. For this, we injected 5-ethynyl-2′-deoxyuridine (EdU) to label proliferating cells **(Figure 3E)**. The endothelial nucleus was stained with ERG1. KV11 only mildly reduced the number of proliferating ECs, as determined by the number of EdU^+^ ERG^+^ nuclei, while EXO_KV11_ showed a stronger reduction effect **(Figure 3F)**. This effect was not due to the EXOs themselves as EXOs alone did not change EC proliferation significantly (**Figure 3F**). qPCR also showed that the expression of key molecular that drive neovascularization such as HIF1α and VEGF were significantly reduced upon EXO_KV11_-treatment, while EXOs alone and KV11 had no clear effect **(Figure 3 G,H)**.

### EXO_KV11_ suppresses vascular leakage in the retina of OIR model mice

**Figure 4:**
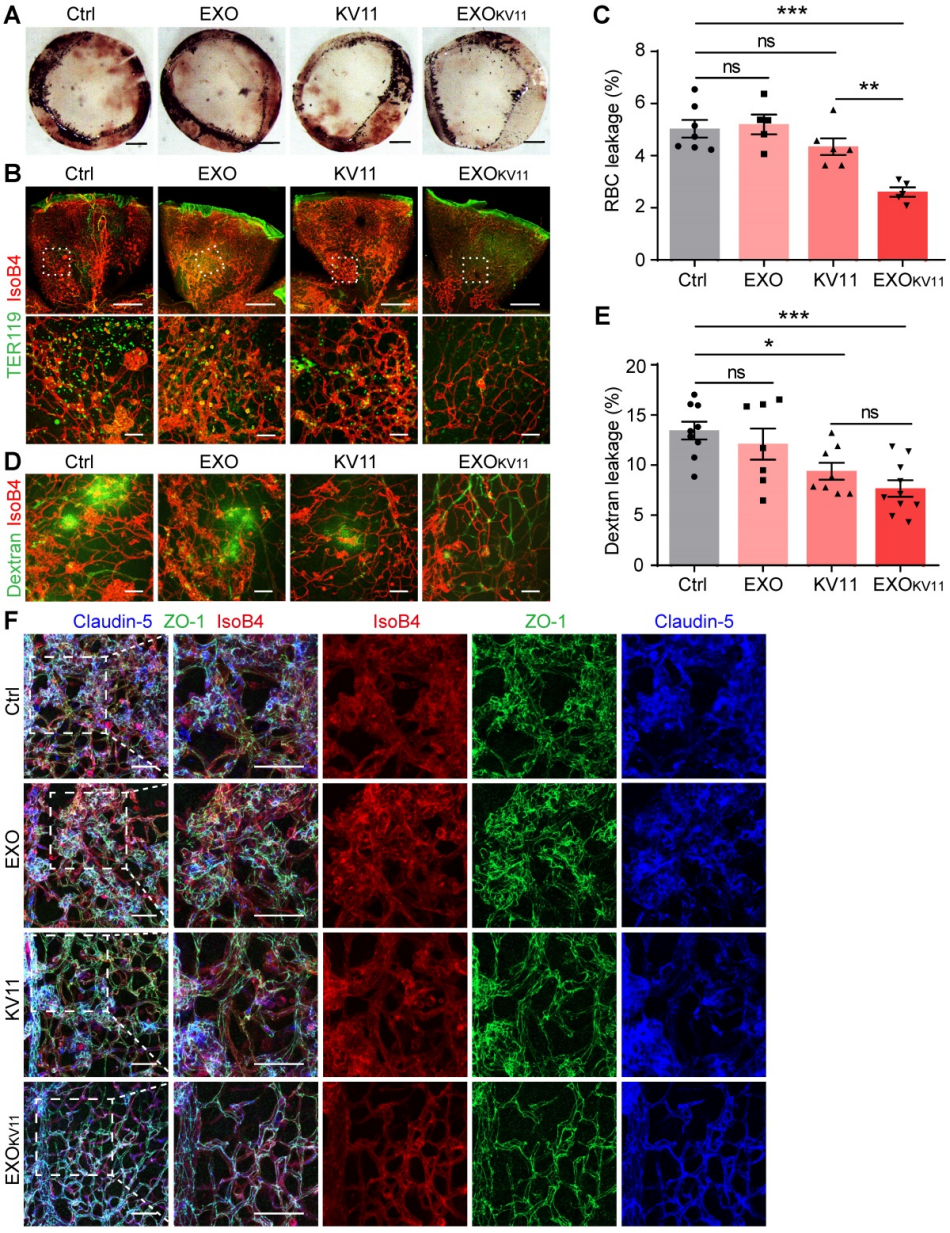
** EXO_KV11_ inhibits vascular leakage in the OIR model.** (A) Images of the inner surface of the OIR retinal cup at P17. Blood island formation indicates hemorrhages in the retinas. (B and D) Representative confocal images of TER119^+^ red blood cell (RBC) leakage (B) and extravasated FITC-conjugated dextran (70 kDa) (D) in flat-mounted retinas from OIR pups with the indicated treatment. (C and E) Quantification of (C) RBC leakage in (B), and (E) dextran leakage in (D) (n = 5 - 9 mice per condition). (F) Representative confocal images of IsoB4-, anti-Claudin-5- and anti-ZO-1-stained vessels in the OIR retinas with indicated treatment.

The abnormal vascular growth in OIR model mice also results in a breakdown of the BRB; thus, severe and extensive retinal hemorrhages were found in the retinas of OIR pups. This was dramatically decreased in EXO_KV11_-treated pups (**Figure 4A**). We further performed immunohistological analysis for TER119 (an erythrocyte marker) and IsoB4. EXO_KV11_ inhibited erythrocyte extravasation, as determined by quantifying TER119^+^ IsoB4^-^ area in the whole-mount retina, while EXO or KV11 alone showed no significant effect (**Figure 4B,C**). In addition, FITC-dextran was injected to determine vessel leakage. Extravasated FITC-dextran was detected in OIR pups, but the degree of leakage was markedly decreased in EXO_KV11_-treated pups** (Figure 4D,E)**. Again, the protective effect of EXO or KV11 was not significant **(Figure 4A-E)**.

Tight junctions, including Claudin-5 and ZO-1 are essential for regulating vascular barrier function and determine vascular permeability 43. In the OIR retina, the Claudin-5 and ZO-1 junction distribution was discontinuous. Strikingly, EXO_KV11_ treatment clearly rescued the junction defects, indicating that EXO_KV11_ treatment protecting vascular leakage by preventing destruction of tight junctions **(Figure 4F)**.

To determine the inflammatory status, we analyzed macrophage infiltration in the whole-mount OIR retina. The number of F4/80^+^ macrophages in both the KV11 and EXO_KV11_ treated group decreased, but the latter had a stronger effect **(Figure S4A,B)**. Consistently, the expression of interleukin-6 (IL-6) and vascular cell adhesion molecular-1(VCAM-1) was markedly reduced by EXO_KV11_ treatment **(Figure S4C,D)**. Thus, these results suggested that EXO_KV11_ limits inflammation in the OIR retina.

### EXO_KV11_ suppresses VEGF-induced vascular leakage *in vivo*

**Figure 5:**
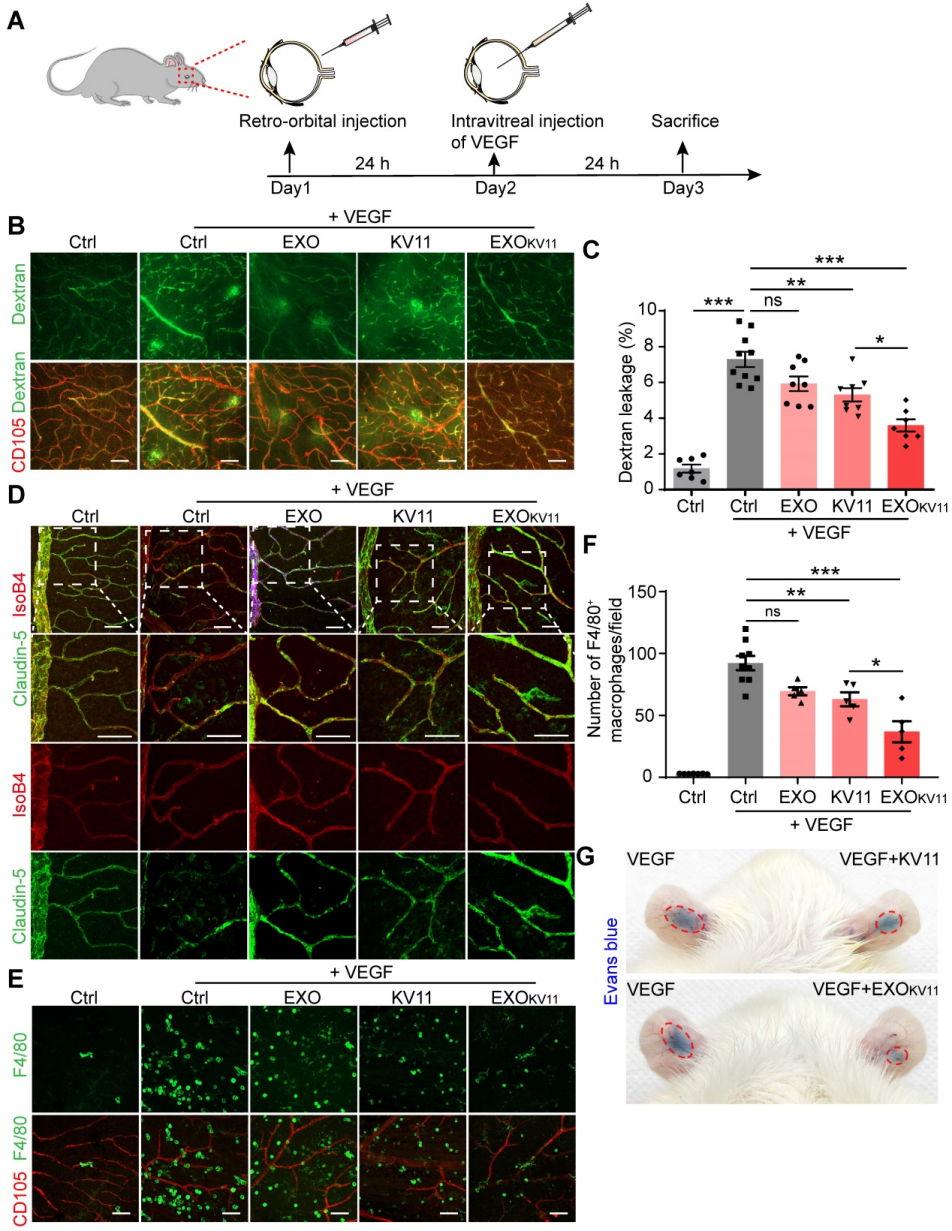
** EXO_KV11_ suppresses VEGF-induced vascular leakage *in vivo*.** (A) Schematic depiction of the pre-treatment procedure in a VEGF-induced vascular leakage model. EXO_KV11_, KV11, EXO, or vehicle was retro-orbitally injected in adult wild type C57BL/6J mice. After 24 h, 100 ng VEGF was intravitreally injected to induce vascular leakage in the retina. The mice were sacrificed for analysis after another 24 h. (B) FITC-dextran was injected and the retinas were harvested at the endpoint. Representative images of flat-mounted retina show extravasated FITC-dextran and CD105^+^ vessels. (C) Quantification of dextran leakage in (B) (n = 7 - 10 mice per condition). (D) Representative confocal images of anti-CD105, anti-Claudin-5-stained retinal vessels in mice treated as in (A). (E) Representative images of F4/80^+^ macrophages (green) and CD105^+^ vessels in retinas treated as in (A). (F) Quantification of macrophage infiltration in (E) (n = 4 - 9 mice per condition). (G) Representative photographs of Evans blue leakage in SD rats ears in an auricular Miles assay with intradermal injection with VEGF, VEGF+KV11 mixture, or VEGF+EXO_KV11_ mixture. The data represent as mean ± SEM, *p < 0.05, **p < 0.01, ***p < 0.001, one-way ANOVA followed by Tukey's multiple comparisons test in (C), (F). Scale bars, 50 µm.

In the mouse OIR model, increased VEGF secretion caused by hypoxia is the major cause of neovascularization and vascular leakage. Thus, we investigated whether EXO_KV11_ could also suppress vascular endothelium activation by VEGF in an *in vivo* model. We first explored whether EXO_KV11_ has prevention effects in VEGF-induced vascular model. For this, EXO_KV11_, KV11, EXO or vehicle was retro-orbitally injected into adult C57BL/6J mice. Twenty-four hours later, 100 ng VEGF was intravitreally injected** (Figure 5A)**. Vascular leakage was visualized by FITC-dextran perfusion. VEGF-induced vascular leakage was strongly suppressed by EXO_KV11_, while KV11 was less effective (**Figure 5B,C**). When we intravitreally injected a VEGF-trap, which is currently being used for treating neovascular retinal disease, we found that it also significantly inhibited VEGF induced vascular leakage in our mouse model, and the effect of retro-orbital injection of EXO_KV11_ was comparable with the effect of intravitreal injection of the VEGF-trap (EXO_KV11_ 50.71% ± 7.18% reduction vs. VEGF-trap 53.09% ± 8.62% reduction) (**Figure 5B,C and Figure S5A,B**). Consistently, VEGF injection affected the organization of Claudin-5, and this was protected by EXO_KV11_ treatment **(Figure 5D)**. We also performed immunofluorescence staining for F4/80 and the endothelial marker CD105. We found that although F4/80^+^ macrophages were abundantly enriched in the retinas of VEGF-treated mice, these macrophages were scarce in EXO_KV11_-treated retinas, suggesting that EXO_KV11_ also limits inflammation in the VEGF-treated retina (**Figure 5E,F**). Again, the effect of retro-orbital injection of EXO_KV11_ was comparable with intravitreal injection of the VEGF-trap (EXO_KV11_ 60.04% ± 8.36% reduction vs. VEGF-trap 62.87% ± 10.0% reduction), while retro-orbital injection of KV11 was less effective (**Figure 5E,F and Figure S5C,D**). By using this pre-treatment model, our results indicated that EXO_KV11_ improved vascular integrity and suppressed inflammation in the VEGF-treated mouse retina.

**Figure 6:**
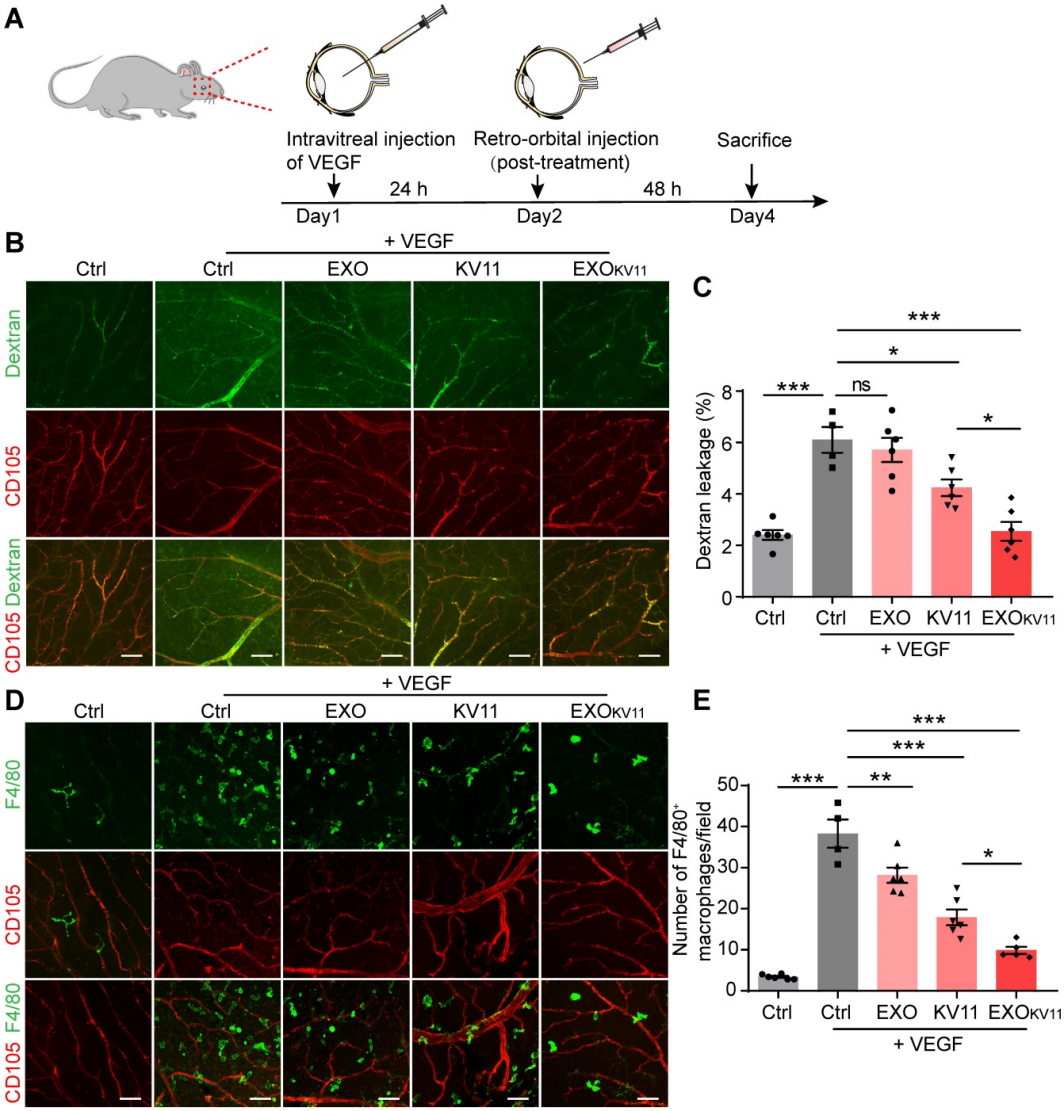
** EXO_KV11_ has therapeutic effect in post-treatment model.** (A) Schematic depiction of the post-treatment procedure in VEGF-induced vascular leakage model. 100 ng VEGF was intravitreally injected to induce vascular leakage in the retina. After 24 h, EXO_KV11_, KV11, EXO, or vehicle was retro-orbitally injected in adult wild type C57BL/6J mice. The mice were sacrificed for analysis after another 48 h. (B) FITC-dextran was injected and the retinas were harvested after treatment. Representative images of flat-mounted retina show extravasated FITC-dextran and CD105^+^ vessels. (C) Quantification of dextran leakage in (B) (n = 4 - 6 mice per condition). (D) Representative images of F4/80^+^ macrophages (green) and CD105^+^ vessels in retinas treated as in (A). (E) Quantification of macrophage infiltration in (D) (n = 4 - 6 mice per condition). The data represent as mean ± SEM. *p < 0.05, **p < 0.01, ***p < 0.001, one-way ANOVA followed by Tukey's multiple comparisons test in (C), (E). Scale bars, 50 µm.

The previous experiments revealed EXO_KV11_ prevent vascular leakage after VEGF injection. Next, we investigated whether it has also therapeutic effect by using a post-treatment model. For this, we injected 100 ng VEGF intravitreally to induce vascular leakage. 24 h later, EXO_KV11_, KV11, EXO or vehicle was retro-orbitally administrated** (Figure 6A)**. Vascular leakage was visualized by FITC-dextran perfusion 48 h after the treatment. Analysis of dextran extravasation showed that in the post-treatment model, EXO_KV11_ significantly inhibited vascular leakage **(Figure 6B,C)**. Similarly, quantification of F4/80^+^ macrophage demonstrated that EXO_KV11_ dramatically reduced macrophage infiltration **(Figure 6D,E)**. Again, KV11 also had an inhibitory effect, but was less effective **(Figure 6B-E)**. Collectively, we showed that EXO_KV11_ treatment is not only useful when given before VEGF injection, but as a post-treatment strategy can also inhibit vascular leakage and limit inflammation. Additionally, an auricular Miles assay showed that EXO_KV11_ reduced VEGF-induced vascular leakage, suggesting a conserved protection mechanism in the mature vasculature (**Figure 5G**).

### Assessment of toxicity in EXO_KV11_-treated mice

**Figure 7:**
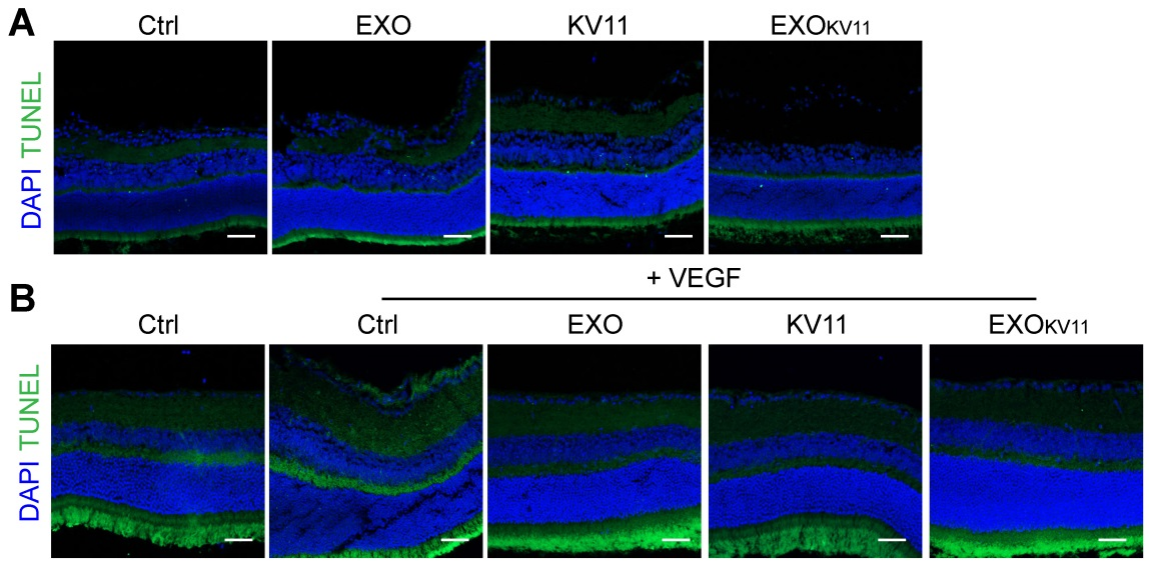
** Assessment of eye toxicity in EXO_KV11_-treated mice.** (A and B) Representative TUNEL assay images of eye sections from saline-treated controls (Ctrl), EXO-, KV11-, or EXO_KV11_-treated mice in the OIR at P17 (A) and pre-treated VEGF-induced vascular leakage model at indicated endpoint(B).

We next examined the toxicity of EXO_KV11_ to the retina as well as the major organs, including the liver, spleen, lung, and kidney. TUNEL assay showed that TUNEL^+^ cells in the retina of the EXO_KV11_ treated mice were not more than those in saline-treated controls in either the OIR model (**Figure 7A**) or the VEGF-induced vascular leakage model (**Figure 7B**). Histological analysis also showed no evidence of* in vivo* toxicity of EXO_KV11_ in either the model (**Figure S6A,B**). The concentrations of the hepatic enzymes alanine aminotransferase (ALT) and alanine aspartate aminotransferase (AST) in the serum of EXO_KV11_-treated mice were also not significantly different from those in saline-treated controls in either model (**Figure S6C-F**), suggesting that EXO_KV11_ was not significantly toxic.

### EXO_KV11_ suppresses VEGF-downstream signaling in ECs

**Figure 8:**
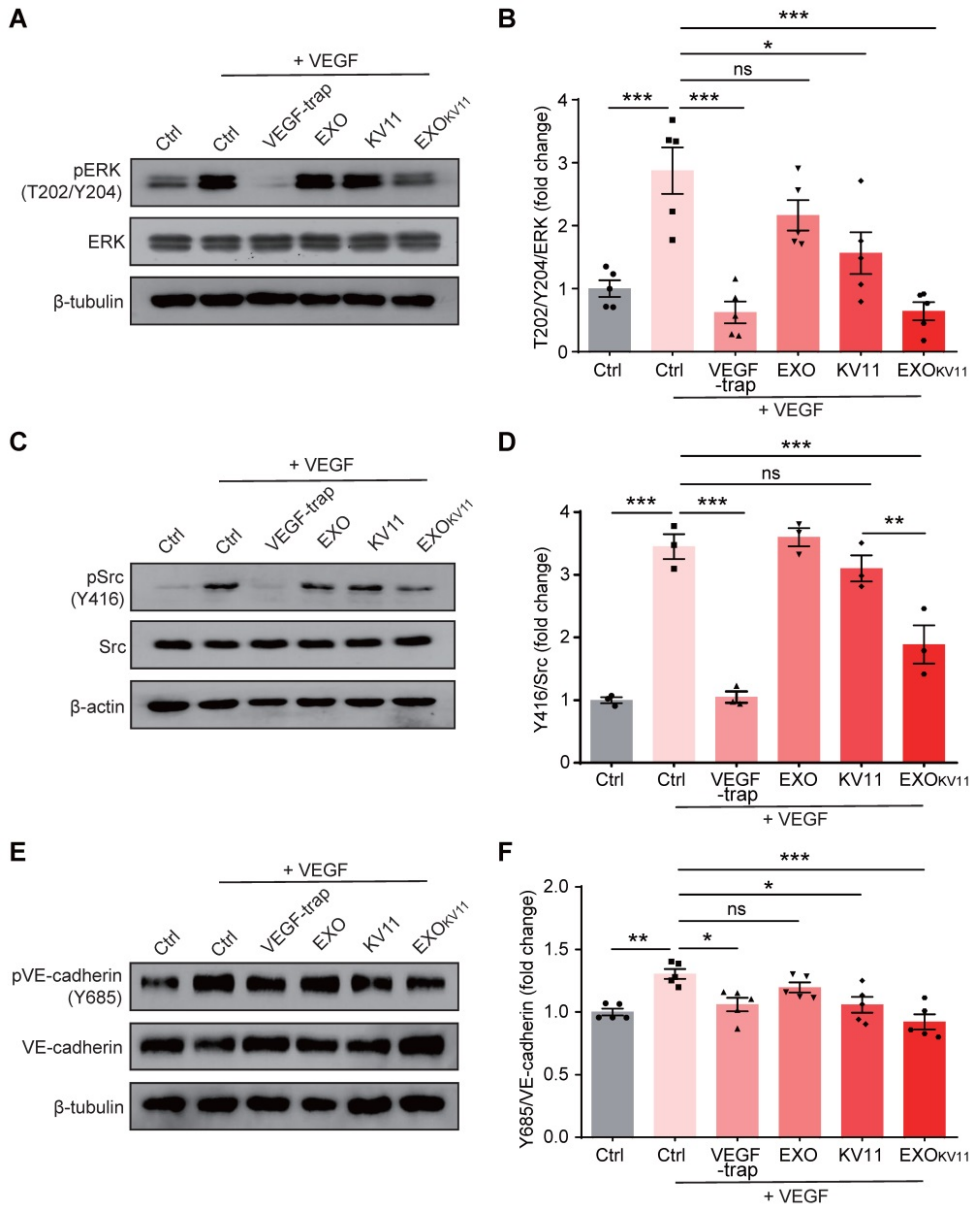
** EXO_KV11_ suppresses VEGF-induced endothelial proliferation and VE-cadherin phosphorylation.** (A, C) Starved HUVECs were pretreated with EXO, KV11, or EXO_KV11_ for 24 h and then stimulated with 25 ng/mL VEGF for 10 min. Representative western blots for pERK (A) and pSrc (C) showing VEGF-downstream signaling. Conbercept (VEGF-trap) was used as a positive control, the pretreatment time was 5 min before VEGF stimulation. 30 μg total protein from each sample was loaded. (E) Starved HUVECs were pretreated with EXO, KV11, or EXO_KV11_ for 24 h and then stimulated with 25 ng/mL VEGF for 15 min. Representative western blot for phosphorylation of VE-cadherin at site Y685 showing how the indicated pretreatment affects VEGF-induced phosphorylation of VE-cadherin at Y685. Conbercept (VEGF-trap) was used as a positive control, the pretreatment time was 5 min. 30 μg total protein from each sample was loaded. (B, D, and F) Quantification of blots shown in (A), (C), and (E); n = 3 - 5 independent experiments. The data represent as mean ± SEM. *p < 0.05, **p < 0.01, ***p < 0.001, one-way ANOVA followed by Tukey's multiple comparisons test in (B), (D), (F).

Although the mechanism of how KV11 inhibits angiogenesis is not clearly understood, it has been shown KV11 suppressed extracellular-signal-regulated kinase (ERK) activation as well as Src activation under VEGF stimulation 31. As ERK is crucial in regulating EC proliferation 44, 45, while Src is involved in regulating cell migration, endothelial junctions, and barrier function 46, we then performed western blot to test whether EXO_KV11_ could also suppress such VEGF-downstream signaling in HUVECs. Consistent with the previous report 31, we indeed observed that KV11 reduced ERK1/2 phosphorylation in response to VEGF stimulation. This effect was more robust in HUVECs pretreated with EXO_KV11_ (**Figure 8A,B**). We also found that EXO_KV11_ significantly reduced Src phosphorylation upon VEGF stimulation, while KV11 showed only a milder reduction (**Figure 8C,D**). VEGF also induces phosphorylation of Y685 of VE-Cadherin, which is involved in vascular permeability 47. Pretreatment with KV11 also suppressed VEGF-mediated Y685 phosphorylation in HUVECs, while EXO_KV11_ showed a stronger reduction (**Figure 8E,F**). Moreover, these effects were not observed in the EXO group, suggesting that the HUVEC-derived EXO itself has no clear effect in blocking VEGF-downstream signaling (**Figure 8A-F**).

### EXO_KV11_ inhibits VEGF-induced EC proliferation, migration, and sprouting* in vitro*

**Figure 9:**
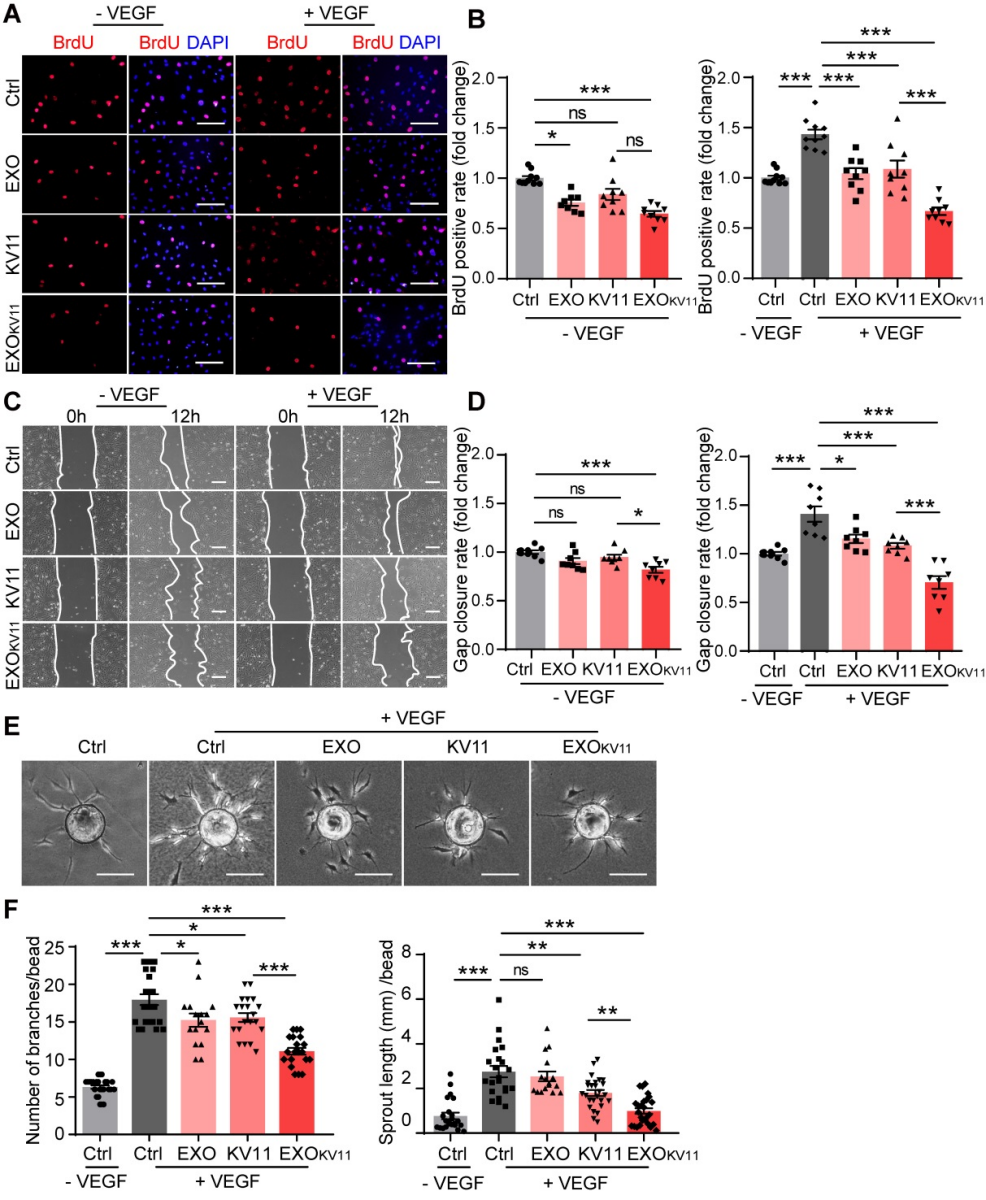
** EXO_KV11_ suppresses VEGF-induced angiogenic effects.** (A) Starved HUVECs were pretreated with EXO, KV11, or EXO_KV11_ and stimulated with VEGF for 24 h. Staining for BrdU incorporation shows proliferating HUVECs. (B) Quantification of BrdU^+^ cells in (A). The proportion of BrdU^+^ nuclei among total DAPI^+^ nuclei was determined and then normalized to the control condition. n > 8 biological repeats were analyzed. (C) Representative bright field images of the scratch migration assay of HUVECs treated as in (A) after 12 h of VEGF stimulation.(D) Quantification of the gap closure from (C). n = 8 biological repeats were quantified, and the results were normalized to the control condition. (E) Representative images of the bead-sprouting assay using HUVECs pretreated with EXO, KV11, or EXO_KV11_ and treated with 50 ng/mL VEGF for 24 h. (F) Quantitative analysis the number of sprouts and total sprouting length in (E). Approximately 20 beads per condition were quantified. The data represent as mean ± SEM. *p < 0.05, **p < 0.01, ***p < 0.001, one-way ANOVA followed by Tukey's multiple comparisons test in (B), (D), (F). Scale bars, 50 µm in (A and C), 200 µm in (E).

Our data indicated that EXO_KV11_ suppressed VEGF-downstream signaling in cultured ECs. To further characterize the EC angiogenesis phenotype after EXO_KV11_ treatment, we performed a set of *in vitro* angiogenesis assays. EXO_KV11_-treated mouse retinas in the OIR model showed reduced proliferation **(Figure 3E)**. To test whether EXO_KV11_ has a direct inhibitory effect on EC proliferation, we performed a 5-Bromo-2-deoxyuridine (BrdU) incorporation assay in HUVECs. In ECs without VEGF stimulation, EXO_KV11_ already showed an inhibitory effect on proliferation, while KV11 alone had no obvious effect. After VEGF stimulation, EXO_KV11_ pretreatment showed a more pronounced inhibitory effect, and the difference between KV11 and EXO_KV11_ became apparent, suggesting that EXO_KV11_ already has an inhibitory effect in the basal state, and this effect was more marked after VEGF stimulation (**Figure 9A,B**).

Expansion of vascular networks also requires EC migration. Therefore, we also tested whether EXO_KV11_ could affect EC migration. For this, we performed a wound scratch assay. In the basal state without VEGF stimulation, while EXOs or KV11 alone did not have a significant effect on EC migration, EXO_KV11_ significantly inhibited wound closure. After VEGF stimulation, KV11 alone showed a significant inhibitory effect on wound closure, while EXO_KV11_ showed a more robust inhibitory effect and the difference was highly significant (**Figure 9C,D**).

We also analyzed EC sprouting *in vitro* using the beads sprouting assay 2. VEGF stimulation significantly increased the number of sprouts and the total sprouting length. EXO_KV11_ treatment successfully reduced both parameters in this assay system (**Figure 9E,F**).

Collectively, these results indicate a strong anti-angiogenic effect of EXO_KV11_ in ECs, especially in response to VEGF stimulus.

## Discussion

In this study, we constructed an EXO-based anti-angiogenic peptide delivery system and assessed its feasibility, safety, and efficacy. We showed that this EXO_KV11_ system targeted the retina blood vessels, and reduced pathological angiogenesis as well as vascular leakage. Importantly, EXO_KV11_ can be efficiently delivered into the retina vasculature by retro-orbital injection, a less invasive procedure than intravitreal injection, which is a commonly used method in the clinic 33-35. Our findings suggest that EXO_KV11_ will work as an effective nanotherapeutic for treating pathological angiogenesis in retinopathy.

KV11 is an 11-amino acid peptide from the KV domain of apolipoprotein(a), a known anti-angiogenic factor 31. KV11 peptide shows anti-angiogenic activity not only* in vitro*, but also *in vivo* in several animal models, including the chicken chorioallantoic membrane model, the mouse corneal micropocket angiogenesis model, and also the mouse xenograft tumor model 31. In the retina, KV11 also shows an anti-angiogenesis effect in the mouse OIR model. However, to achieve this, KV11 must be injected intravitreally and the treatment requires repeated injections 32. In this study, therefore, we sought a better solution.

EXOs have attracted considerable attention as drug delivery vehicles due to their low immunogenicity and high safety profile. They are also relatively stable with a longer half-life in the blood circulation, as they avoid opsonins, coagulation factors, complement, and phagocytosis by circulating monocytes and macrophages 48. Furthermore, the membrane structure of EXOs enables them to cross the cell membrane system and to be delivered into the recipient cell. Although the mechanisms of EXO uptake and of components delivery into acceptor cells, whether they involve membrane fusion or endocytosis and whether the process has cell type targeting specificity, are still incompletely characterized 49, several studies have suggested that the surface components on EXOs and acceptor cells, such as integrins, tetraspanins, and phosphatidylserine 50-53, probably influence EXO target recognition and cellular uptake by target cells. Physiologically relevant specific targeting or homing remains to be achieved. Notably, however, some studies have already indicated that EXOs have the ability to target their parent cells and to serve as selective vehicles. For example, it has been shown that the U251 glioblastoma cells derived EXOs exhibit higher internalization efficiency in U251 cells than astrocyte derived EXOs 54. It was also shown that human placental mesenchymal stem cells (MSCs) derived EXOs preferentially go to the cell type of origin 55. Other studies also indicated that EXOs derived from certain tumor cells have the ability to home to their parent tumors 56, 57. Thus, considering EXOs natural homing properties, we chose ECs derived EXOs as carriers for blood vessel delivery in this study, as they would be more effective at homing to where they were secreted from than to other cells.

Using an anchor peptide that we had previously identified 30, therapeutic amounts of KV11 were loaded onto EXOs and elicited a functional inhibitory effect on pathological retinal angiogenesis. Importantly, in the OIR model, instead of two intravitreal injections of KV11 as previously reported 32, a single retro-orbital injection of EXO_KV11_ efficiently rescued the vascular defects without any detectable toxicity, indicating that the EXO is an efficient delivery vehicle for treating vascular dysfunction in the retina.

Functioning as carriers of small bioactive molecules, EXOs have emerged as important players in cell-cell communication. It has been shown that EXOs derived from different cell types can deliver pro- and anti-angiogenic factors including proteins, mRNA, and microRNAs into ECs and can thus regulate angiogenesis. For instance, during tumor progression, EXOs secreted from tumor cells interact with ECs and stimulate angiogenesis in multiple tumor models 58-60; this may be a critical step for tumor angiogenesis and metastasis. ECs also secrete EXOs. Interestingly, under different patho/physiological conditions, ECs secrete EXOs that have different contents and that exert different biological functions. For example, Delta-like 4 is incorporated into endothelium-derived EXOs, which then signal to neighboring ECs, inhibit Notch signaling, and increase capillary formation 61. It was also shown that extracellular vesicles released by ECs contain matrix metalloproteinases that stimulate ECs to form capillary-like structures 62. On the other hand, another study revealed an anti-angiogenic effect of extracellular vesicle(s), namely that endothelium-derived microparticles can also impair angiogenesis by inhibiting EC proliferation and increasing apoptosis 63. Notably, under pro-angiogenic stimulation by factors such as VEGF, FGF2, or high glucose, EC-secreted EXOs may contain different amounts of particular contents, which will further alter their angiogenic properties 62, 64. In our study, we observed that HUVEC-derived EXOs alone slightly reduced EC proliferation and migration *in vitro*, but this effect was not observed in *in vivo* models. The EXOs that we used were derived from HUVECs cultured in normal endothelial growth medium without any stimulation; therefore, if we use VEGF- or pro-inflammatory factor-treated HUVECs, we may obtain different results as such cells could secrete EXOs with different angiogenic properties.

Although this study focused on retinal angiogenesis, it will also be interesting to test the efficacy of peptide-loaded EXOs in treating pathological angiogenesis in other disease models. It is worth noting that the wet form of age-related macular degeneration (wet AMD) characterized by choroidal neovascularization, is the leading cause of vison loss among elderly persons in developed countries 65. The pathogenesis of wet AMD is due to the excessive production of VEGF from hypoxic cells in the retino-choroidal complex, which further leads to the growth and abnormal leakage of immature blood vessels. As our results showed that EXO_KV11_ could inhibit vascular leakage and limit the F4/80 infiltration in VEGF-treated retinas, further studies would be required to test whether EXO_KV11_ could also be the potential treatment for wet AMD. Angiogenesis is also essential for tumor growth and metastasis. During solid tumor progression, tumor vessels are also highly proliferative and functionally abnormal, with high permeability. These features facilitate intravasation and dissemination of tumor cells. As EXO_KV11_ suppressed EC proliferation and repaired the barrier leakiness, thus, it would be interesting to test whether EXO_KV11_ can also be used to suppress tumor angiogenesis and improve vascular integrity, as current anti-angiogenic therapy prolongs the survival of patients by only a limited time due to therapy escape or resistance.

In the OIR retina, two types of angiogenesis occurred after the pups were returned to ambient air: revascularization and neovascular tuft formation. Revascularization is a physiological process for repairing the injured retina in which normal blood vessels are formed in the ischemic area. In this study, we observed no difference in the avascular area, suggesting that revascularization is not improved by EXO_KV11_ treatment. This indicates that this system would not ameliorate ischemic disease. Further studies are required to search for a peptide that will yield benefits for revascularization. Notably, we have successfully loaded two peptides with distinctive functions onto EXOs in our previous work 30. Thus, by using this ocular delivery system, it will be interesting to test whether we can achieve better therapeutic outcomes if we load more types of peptides with different biological functions.

In this study, we established a system that uses EXOs as carriers to deliver therapeutics into the ocular region for treating proliferative retinopathy. Our study highlights three unique advantages of EXO-based therapy: (i) the EXO therapeutic system is relatively stable with safety; (ii) EXOs exhibit high efficacy for treating pathological angiogenesis; and (iii) instead of intraocular injection, which is a commonly used method for ocular drug delivery, a less invasive route can be used for EXO-based therapy. Mechanistically, we demonstrated that EXO_KV11_ did not alter the anti-angiogenic property of KV11, but improved the delivery efficiency and achieved better therapeutic efficacy. A limitation of this study is that although our study clearly demonstrated the protective effect of EXO_KV11_ in OIR model and VEGF-induced retinal vascular leakage model, a long-term test could not be applied in such models. Further studies by using animal models, which are suitable for long-term treatment, would be more insightful. Furthermore, as this study used mouse as an experimental model, more extensive study will need to be performed prior to clinical deployment. Overall, our study provides a novel approach and has proved the feasibility of this approach for the future development of clinical translation for therapy.

## Methods Details

### Animals

All study protocols involving the use of animals were approved by the Institutional Animal Care and Use Committee of Tianjin Medical University. Adult wild type C57BL/6J and CD-1 mice, neonatal C57BL/6J mice with mothers were purchased from Model Animal Research Center of Nanjing University. For IVIS imaging and subsequent biodistribution testing, albino CD-1 mice were used. For Miles assay, Sprague Dawley (SD) rats were used. For other experiments, C57BL/6J mice were used.

### Cell Culture

Human Umbilical Vascular Endothelial Cells (HUVECs) were cultured as previously described 66 and were used from passage 2 to passage 6. For exosomes isolation, all cells were cultured in exosome-free normal growth medium (removed possible FBS-contained exosomes by centrifugation at 100,000×g for 16 h at 4 °C). For stimulation experiments, cells were starved overnight in growth factor free M199 medium (Gibco®, Life Technologies, US) supplemented with 2% FBS, 100 U/mL penicillin and 100 µg/mL streptomycin.Human retinal microvascular endothelial cells (HRMECs) were cultured in endothelial cell medium (ECM) containing 5% FBS, 1% endothelial cell growth supplements and 1% antibiotic solution (P/S). All cells were used from passage 2 to passage 6.

### Exosome isolation

Exosomes were isolated as previously described 67. Briefly, cell culture medium was sequentially centrifuged at 1000×g for 10 min, followed by 10,000×g for 30 min. The supernatant was collected and filtered with a 0.22 µm filter (Millex, Germany), followed by ultracentrifugation at 100,000×g for 1 h. Exosome pellets were washed in a large volume of PBS and recovered by centrifugation at 100,000×g for 1 h and was resuspended in sterile PBS for the following experiments. The protein concentration of isolated exosomes was quantified by using the Bradford assay kit (Sangon Biotech, China).

### Exosome characterization

To detect the expression of exosomes associated markers, exosomes pellets and cultured cells were lysed in RIPA lysis buffer containing 1X protease inhibitor. Proteins (50 μg for both exosome and cell lysate) were subjected to 12% SDS-polyacrylamide gel electrophoresis and transferred to a polyvinylidene difluoride membrane (Millipore, Germany). Specific antibodies used for detect exosome associated markers include Alix (3A9) (1:1000, 2171, Cell Signaling Technology, US), CD63 (1:200, sc-5275, Santa Cruz, US), CD81 (1:200, sc-166029, Santa Cruz, US). Cytochrome C (D18C7) (1:1000, 11940, Cell Signaling Technology, US) was used to determine mitochondria contamination.

The size distribution of HUVECs derived exosomes was measured by Nanosight NS300 (Malvern, UK). The morphology of exosomes was visualized by a high-resolution transmission electron microscope (TEM, Hitachi HT7700, Japan). The procedure of exosome staining prepared for TEM was performed as previously described 68.

### Peptides conjugation

The KV11 sequence was YTMNPRKLFDYK; the KV11-CP05 sequence was YTMNPRKLFDYKCRHSQMTVSRL. KV11 and KV11-CP05 were synthesized as a single fusion peptide via peptide bond without spacer by ChinaPeptides (Shanghai, China) with 98% of purity.

### Peptide binding and cellular uptake *in vitro*

To test the binding affinity of KV11-CP05 on EXO, 5 μg exosomes were preincubated with FITC-labeled KV11-CP05 (20 μg) in a 1.5 mL eppendorf tube under rotation at 4 °C for 6 h, followed by washing with PBS at 7500×g for 30 min for five times in 2 mL ultracentrifuge tubes and filtration with 100-kDa diafiltration tubes (Millipore, German) to remove unbound peptides. Final solution was collected by reversing the tubes and centrifugating at 1500×g for 30 min and analyzed with flow cytometry.

To test the cellular uptake of exosomes by HUVECs and HRMECs, isolated exosomes were labeled with DiR (Invitrogen, US) following the manufacturer's instruction. 10 μg/mL labeled exosomes were incubated with HUVECs or HRMECs for 24 h in exosomes free medium. Cells were then washed with cold PBS and fixed for 30 min at RT with 4% PFA and nuclei were counterstained with DAPI (Invitrogen, US). DiR and DAPI were imaged on a confocal microscope (LSM800, Carl Zeiss, Germany).

To determine the delivery efficiency of EXO_KV11_ in HUVECs and HRMECs, DiR-labeled exosomes (5 μg) were incubated with FITC-labeled KV11-CP05 (20 μg) or FITC-labeled KV11 (20 μg) at 4 °C for 6 h. Subsequently, peptides/exosomes mixture or peptide-exosome complexes were incubated with HUVECs or HRMECs in 24-well plate for 24 h or 48 h. Cells were washed with cold PBS and fixed for 30 min at RT with 4% PFA and nuclei were counterstained with DAPI. Images were obtained by confocal microscope. To compare peptide delivery efficiency in HUVECs and HRMECs, cells of each group were harvested and was analyzed with flow cytometry (FACSVerse, BD, US).

### Ocular distribution

FITC-labeled KV11 peptide and KV11-CP05 were synthesized by ChinaPeptides (Shanghai, China). To examine the ocular distribution of peptide, KV11 or EXO_KV11_ were administered retro-orbitally or intravitreally into CD1 mice. 50 μg FITC-KV11 and EXO_KV11_ (50 μg FITC-KV11-CP05 and 25 μg EXO were incubated for 6 h at 4 °C before injection) were dissolved in saline solution (0.9% NaCl) and injected retro-orbitally. 2 μg FITC-KV11 and EXO_KV11_ (2 μg KV11-CP05 and 1 μg EXO were incubated for 6 h at 4 °C before injection) in saline solution (0.9% NaCl) and injected intravitreally. After indicated time points (6 h, 12 h for retro-orbital injection or intravitreal injection), the mice were perfused with cold PBS to wash out free peptides and exosomes in the circulation. Eyes were then harvested for imaging with IVIS spectrum (PE, Waltham, MA, US). The fluorescence intensity was measured by using the ROI tool of Living Image software (V4.2). Briefly, the region of the eyes was selected by using the ROI tool and the fluorescence intensity was measured. After subtracting the background fluorescence, the data were applied for analysis.

To detect the distribution of FITC-labeled peptides in retinal sections, eyes from each group were enucleated in PBS after mice were sacrificed. The eyes were fixed in 4% PFA for 1 h at 4 °C. Afterwards, they were transferred to 30% sucrose/PBS at 4 °C overnight and subsequently embedded in optimal cutting temperature compound (OCT) (Sakura, Japan) and frozen at -80 °C. Serial 15 μm-thick sections were cut using a cryostat (CM1950, Leica, Germany). Cryosections were washed in PBS for 10 min, permeabilized in 0.3% TritonX-100 PBS for 15 min and then blocked in 2% BSA, 0.3% TritonX-100 PBS for 1 h. Cryosections were then incubated with IsolectinGS-IB4 (Alexa Fluor 568-conjugated, 1:200, I21413, Invitrogen, US) for 2 h, that was used for labeling the blood vessels. Images were collected on a confocal microscope (LSM800, Carl Zeiss, Germany). To quantify the abundance of FITC labeled peptides delivered to the blood vessels, the fluorescence intensity of FITC was analyzed in isolectinB4 positive area by using image J software.

### Oxygen induced retinopathy (OIR) model

Oxygen induced retinopathy model was performed as previously described 54. Briefly, neonatal C57BL/6J mice with the nursing mother at day P7 were exposed to hyperoxia (75% O_2_) for 5 days. Mice returned to room air at P12. For applying treatment, 25 μg EXO, 50 μg KV11 and EXO_KV11_ (50 μg KV11-CP05 and 25 μg EXO were incubated for 6 h at 4 °C before injection) were dissolved in saline solution (0.9% NaCl) and injected retro-orbitally at P12. The retinas were collected at P17 for analysis.

### VEGF induced retina vascular leakage model

In pre-treatment model, adult wild type C57BL/6J mice were pretreated retro-orbitally with 50 μg KV11, 25 μg EXOs or EXO_KV11_ (50 μg KV11-CP05 and 25 μg EXO were incubated for 6 h at 4 °C before injection) in 50 μL saline solution (0.9% NaCl). In VEGF-trap treated group, 15 μg VEGF-trap (10 mg/mL Conbercept Ophthalmic Injection, KANGHONG, China) was pretreated intravitreally by a 33-gauge beveled needle. 1 μL VEGF164 protein (100 ng/μL) was injected intravitreally 24 h after pretreatment using a 33-gauge beveled needle. Mice were sacrificed and retinas were collected for analysis after another 24 h.

In post-treatment model, 1 μL VEGF164 protein (100 ng/μL) was injected intravitreally. After 24 h, mice were treated retro-orbitally with 50 μg KV11, 25 μg EXO or EXO_KV11_ (50 μg KV11-CP05 and 25 μg EXO were incubated for 6 h at 4 °C before injection) in 50 μL saline solution (0.9% NaCl). After 48 h post-treatment, the retinas were collected for analysis.

### Immunofluorescence

For flat-mounted retinas, eyes were enucleated from mice and fixed in 4% PFA for 1 h at 4 °C. Retinas were dissected, washed with PBS and permeabilized with PBS containing 1% TritonX-100 overnight at 4°C then blocked in PBS containing 2% BSA, 0.3% TritonX-100 for 12 h at 4 °C. After blocking, for visualization of retinal vasculature in OIR models, flat-mounted retinas were stained with isolectinGS-IB4 (1:100) for 2 h at RT. For IF staining of red blood cells, blood vessels, macrophage and microglia, flat-mounted retinas were incubated in blocking solution with following primary antibodies at 4 °C overnight: rat anti-TER119 monoclonal (1:200, MAB1125, RD, US); rat anti-CD105 monoclonal (1:200, MAB1320, RD, US); rabbit anti-F4/80 monoclonal (1:200, 30325S, Cell Signaling Technology, US); mouse anti-Claudin-5 monoclonal (1:200, 35-2500, Invitrogen, US) and rabbit anti-ZO-1 polyclonal (1:300, 61-7300, Invitrogen, US). After washing, the retinas were incubated with corresponding secondary antibodies (1:300, Jackson ImmunoResearch, US) for 2 h at RT. Flat-mounted retinas were analyzed using a confocal fluorescence microscope (LSM 800, Carl Zeiss, Germany).

### Analyses of vascular leakage and perfusion

Vascular leakage was analyzed by intracardial injection performed as previously described 69. In brief, 31-G needle was positioned above the heart 2 mm parasternal to the left at a virtual line connecting both armpits, in caudal and lateral angles of 30° and 10°, 50 μL, warm PBS containing FITC-conjugated dextran (25 mg/mL, 70 kDa, FD70S, Sigma-Aldrich, US) were injected. After 10 min of circulation, eyes were enucleated and fixed in 4% PFA for 1 h at 4 °C. In OIR model, retinas were then dissected, washed with PBS and permeabilized with PBS containing 1% TritonX-100 overnight at 4 °C and stained with isolectinGS-IB4 (1:100) for 2 h at RT, and flat-mounted in fluorescent mounting medium (Fluoromount-G®, 0100-01, SouthernBiotech, US) after several washes. In VEGF induced retina vascular leakage model, retinas were incubated in blocking solution with the primary antibody rat anti-CD105 monoclonal (1:200, MAB1320, RD, US) at 4 °C overnight after permeabilization and blocking. Then corresponding secondary antibody was used for 2 h at RT. Leakage was observed and imaged on a confocal fluorescence microscope (LSM800, Carl Zeiss, Germany). FITC-conjugated dextran was measured as dextran area outside the vessels divided by total measured area of retina by using image J software.

### Morphometric analyses

Morphometric analyses of the retinas were performed using Image J software and Adobe Photoshop software. To determine the amount of regression and neovascularization, the number of pixels in the avascular regression area and neovascular area was measured using the Lasso tool of Adobe Photoshop software as described 54, and divided by the number of pixels in the total retinal area and presented as a percentage. RBC leakage was measured as TER119-stained area outside the vessels divided by total measured area of retina as previously described 70. Macrophage infiltration was measured as the total number of F4/80^+^ macrophage in the whole retina.

### EdU injection in pups

EdU (E6032, US EVERBRIGHT INC., China) was injected at a concentration of 50 μg/g body weight into pups at P17 in OIR model 2.5 h before sacrifice. Eyes were enucleated from pups and fixed in 4% PFA for 1 h at 4 °C, then were transferred to 30% sucrose/PBS at 4 °C overnight and subsequently embedded in optimal cutting temperature compound (OCT) (Sakura, Japan) and frozen at -80 °C. Afterwards, sagittal cryosections of the eyes were prepared. EdU^+^ cells in the cryosections of retinas were detected by using YF® 488 Click-iT EdU Stain Kits (C6033, US EVERBRIGHT INC., China), according to the manufacturer's instructions. ECs were counterstained with ERG (1:400, ab92513, Abcam, UK) overnight at 4 °C, isolectinGS-IB4 (1:200) and corresponding secondary antibody (1:300, Jackson ImmunoResearch, US) for 2 h at RT. The numbers of EdU^+^ERG^+^ cells were counted from five sagittal eye sections. To ensure that similar areas were used for doing quantification for each eye, we always use sections intersect the optic nerve area to maintain consistency.

### RNA extraction and quantitative real-time PCR analysis

Eyes were enucleated from pups at P17 in OIR model. Retinas were dissected and homogenized in TRIzol® reagent (Invitrogen, US). RNA samples were reverse-transcribed to complementary DNA (cDNA) using TransScript One-Step gDNA Removal and cDNA Synthesis SuperMix (TransGen, China). qPCR was performed using *PerfectStart*^TM^ Green qPCR SuperMix (TransGen, China) and was processed with QuantStudio 5 Real-Time PCR system (Applied Biosystems, US). β-actin was used as internal control. All qPCR results were obtained from at least 5 biological repeats. The primers used in this study were: *Hif1a* Forward, 5'-ACCTTCATCGGAAACTCCAAAG-3'; *Hif1a* reverse, 5'-CTGTTAGGCTGGGAAAAGTTAGG-3'; *Vegfa* forward, 5'-GCACATAGAGAGAATGAGCTTCC-3'; *Vegfa* reverse, 5'-CTCCGCTCTGAACAAGGCT-3'; *Il6* forward, 5'-TAGTCCTTCCTACCCCAATTTCC-3'; *Il6* reverse, 5'-TTGGTCCTTAGCCACTCCTTC-3'. *Vcam1* forward, 5'-AGTTGGGGATTCGGTTGTTCT-3'; *Vcam1* reverse, 5'- CCCCTCATTCCTTACCACCC-3'; *Actb* forward, 5'-GGCTGTATTCCCCTCCATCG-3'; *Actb* reverse, 5'-CCAGTTGGTAACAATGCCATGT-3'.

### *In vitro* EC proliferation assay

*In vitro* BrdU incorporation was performed as described 71. Briefly, 2×10^4^ HUVECs were cultured in collagen coated coverslips in 24-well plates and pretreated with EXO, KV11 or EXO_KV11_ (KV11-CP05 and EXO were incubated for 6 h at 4 °C before treatment) at concentration of 12.5 μg/mL EXO and 25 μg/mL peptide for 24 h in starvation medium. Afterwards, cells were treated with or without VEGF (50 ng/mL) for 24 h. BrdU (10 μM, B5002, Sigma-Aldrich, US) was subsequently added and incubated for 4 h at 37 °C. Cells were fixed in 4% PFA/PBS for 20 min and permeabilized and blocked in PBS containing 2% BSA, 0.3% TritonX-100 for 30 min at RT. Unmasking was done by adding ice-cold 0.1M HCl for 20 min and 2 M HCl for 30 min continued. Then neutralization with sodium borate buffer (0.1 M Na_2_B_4_O7 in water, PH8.5) for 15 min was done at RT prior to primary antibody incubation. An anti-BrdU antibody (1:250, ab6326, Abcam, UK) was incubated in blocking solution overnight at 4 °C and corresponding secondary antibody (1:400, Alexa Fluor 594-conjugated, Jackson ImmunoResearch, US) was incubated for 2 h at RT. Nuclei were counterstained with DAPI (1:1000, D1306, Invitrogen, US). Images were obtained using a fluorescence microscope (BX51, Olympus, Japan). N > 8 biological repeats were analyzed. Quantification was done blind to the experimental condition.

### Scratch assay

To analyze the EC migration, 2×10^5^ HUVECs were cultured in collagen coated in 6-well plates and pretreated with EXO, KV11 or EXO_KV11_ (KV11-CP05 and EXO were incubated for 6 h at 4 °C before treatment) at concentration of 12.5 μg/mL EXO and 25 μg/mL peptide for 24 h in starvation medium. A wound was made by scraping the cell monolayer with a 10 μL pipette tip, and cells were stimulated with or without VEGF (50 ng/mL). Pictures were acquired at time-point zero and 12 h after incubation at 37 °C. The percentage of wound closure between 0 h and 12 h was analyzed with Image J software. N = 8 biological repeats were quantified.

### Fibrin gel bead sprouting assay

Fibrin gel bead sprouting assay was performed as previously described 2. Briefly 1×10^5^ HUVECs were seeded in collagen coated 6-well plates and pretreated with EXO, KV11 or EXO_KV11_ (KV11-CP05 and EXO were incubated for 6 h at 4 °C before treatment) at concentration of 12.5 μg/mL EXO and 25 μg/mL peptide for 24 h in starvation medium. Cytodex 3 microcarrier beads (GE Healthcare) were coated with pretreated HUVECs (mixed at 200 cells per bead), and embedded in 2 mg/mL fibrin gels in 24-well plates by mixing 2 mg/mL fibrinogen (Sigma-Aldrich, US) in PBS, 0.625 units/mL thrombin (Sigma-Aldrich, US) and 0.15 units/mL aprotinin (Sigma-Aldrich, US). Beads were cultured in starvation medium in the presence or absence of 50 ng/mL VEGF. After 24 h, the culture beads were fixed with 4% PFA for 15 min at RT. Images were taken with an inverted fluorescence microscope (Ti2-U, Nikon, Japan) and sprout number and length were analyzed by Image J software. Approximately 20 beads per condition were quantified.

### Auricular miles assay

Auricular miles assay was performed as previously described 72. Briefly, Evans Blue dye (45 mg/kg) was injected intravenously. After 10 min, rats were anesthetized and intradermally injected with 200 ng recombinant VEGF in 20 μL PBS (vehicle) in the left ear, and with recombinant VEGF and KV11(12.5 μg) or EXO_KV11_ (12.5 μg KV11-CP05 and 6.25 μg EXO were incubated for 6 h at 4 °C before treatment) in 20 μL PBS in the contralateral ear. Evans blue extravasation was photographed 3 h after EB injection.

### VEGF downstream signaling analysis

To detect the VEGF downstream signaling, 2×10^5^ HUVECs were pretreated with EXO, KV11 or EXO_KV11_ (KV11-CP05 and EXO were incubated for 6 h at 4 °C before treatment) at concentration of 12.5 μg/mL EXO and 25 μg/mL peptide for 24 h in starvation medium. In VEGF-trap (conbercept) treated wells, cells were pretreated with 10 µg conbercept for 5 min before VEGF stimulation. HUVECs were then stimulated with VEGF (25 ng/mL, R&D systems, US). Cells were washed with ice cold PBS and lysed in cold RIPA lysis buffer supplemented with 1X protease inhibitor cocktail and 1X phosphatase inhibitor (Roche, Switzerland). Proteins (30 μg for each sample) were subjected to 10% SDS-polyacrylamide gel electrophoresis and transferred to a polyvinylidene difluoride membrane (Millipore, Germany). Target proteins were detected by specific primary antibodies including Src (36D10) (1:1000, 2109, Cell Signaling Technology, US), Src-Phospho (Y416) (1:1000, AP0452, ABclonal, China), Erk1/2 p44/42 (1:1000, 9102, Cell Signaling Technology, US), Erk1/2 phospho-p44/42 (T202/Y204) (1:1000, 9106, Cell Signaling Technology, US), β-actin (1:1000, 3700, Cell Signaling Technology, US) or β-tubulin (1:1000, UM4003, Utibody, China)-were used as loading control.

The bound primary antibody was detected by horseradish peroxidase-conjugated goat anti-mouse IgG (1:3000, 7076, Cell Signaling Technology, US) and goat anti-rabbit IgG (1:3000, 7074, Cell Signaling Technology, US), respectively. The ECL western blotting analysis system (Millipore) was applied. Quantification of WB signal was done by Image J software. Proteins expression values were divided by their β-actin or β-tubulin, respectively. Each experiment was performed at least three times.

### Toxicity assessment

Toxicity assessment was performed at P17 in OIR model and endpoint in pre-treated VEGF induced vascular leakage model.

Terminal deoxynucleotidyl transferase mediated dUTP nick end labeling (TUNEL) assay was used to examine apoptotic cells in retinas. Mice were sacrificed after indicated endpoint in each model and eyes from each group were enucleated in PBS. The eyes were fixed in 4% PFA for 1 h at 4 °C. Afterwards, they were transferred to 30% sucrose/PBS at 4 °C overnight and subsequently in OCT (Sakura, Japan) and frozen at -80 °C. Serial 15 μm-thick sections were cut using a cryostat (CM1950, Leica, Germany). TUNEL assay was performed using a FragEL^TM^ DNA Fragmentation Detection Kit (QIA39, Millipore, US) according to the manufacturer's instructions.

Routine H&E staining was used to examine the overall major organs morphology, including liver, spleen, lung and kidney from mice in OIR model and VEGF induced retina vascular leakage model. Organs were fixed with 4% formaldehyde overnight at RT, embedded in paraffin, and sectioned to 6-μm thickness. Images were obtained using a microscope (Ti2-U, Nikon, Japan).

To analyze the serum biochemistry, mouse blood was taken immediately after sacrifice and centrifuged at 1,500×g for 30 min and stored at -80 °C. Analysis of alanine aminotransferase (ALT) and aspartate aminotransferase (AST) were performed using AST/GOT KIT and ALT/GPT KIT (BioSino, China) and read by Microlab 300 (Vital, Holland).

### Statistical analysis

Results were expressed as the mean ± SEM. To calculate statistical significance, the Mann-Whitney test or student's t test (two-tailed) (when comparing two groups) or one-way ANOVA followed by Tukey's multiple comparisons test (when comparing three or more groups) were used. *P value* < 0.05 were considered significant. All calculations were performed using Prism software.

## Supplementary Material

Supplementary figures.
