# Supplementary material for: Exosome-mediated delivery of an anti-angiogenic peptide inhibits pathological retinal angiogenesis
Source: Theranostics. 2021 Mar 5;11(11):5107–26. doi: 10.7150/thno.54755 (PMC8039955; doi:10.7150/thno.54755)
Supplement: Supplementary file 1 — Supplementary figures. [file thnov11p5107s1.pdf]

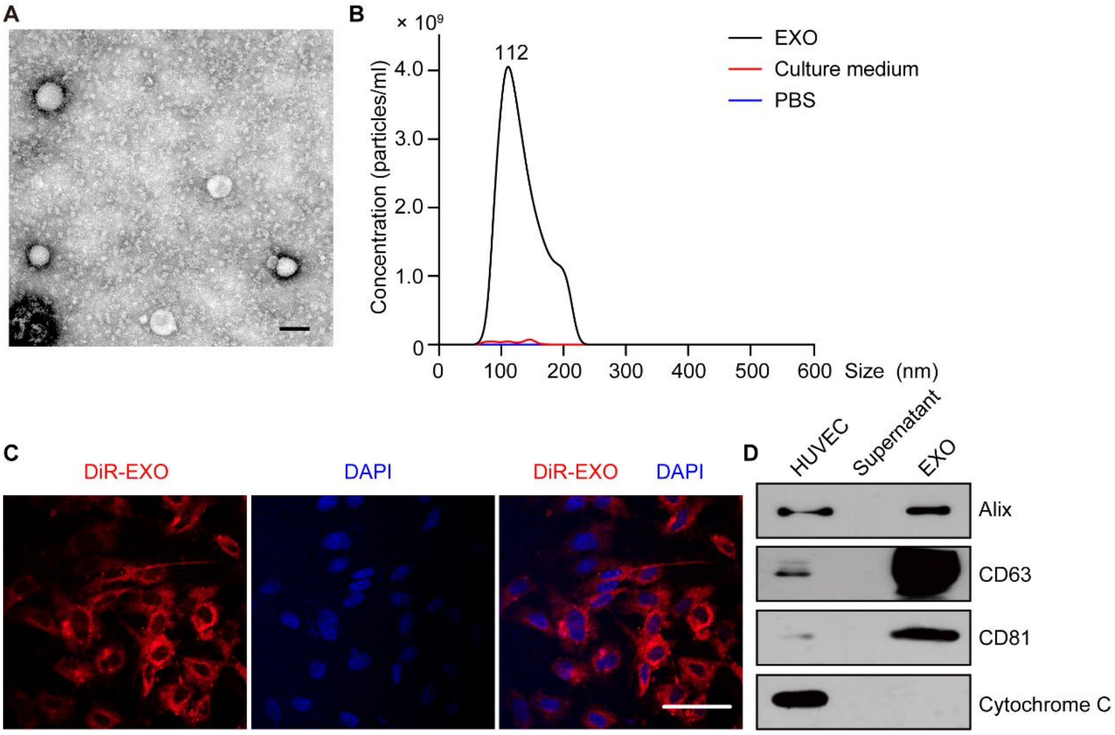

Figure S1. Characterization of EXOs derived from HUVECs.

(A) Representative transmission electron microscopy image of HUVEC-derived EXOs. Scale bar, 100 nm.

(B) Nanosight analysis of the size distribution of EXOs derived from HUVECs (black), particles isolated from equal volume of fresh culture medium (red) and PBS blank control (blue).

(C) Exosomes labeled with DiR were added to cultured HUVECs. Confocal images show the cellular uptake of exosomes into HUVECs after 24 h of incubation. Scale bar, 25 μm.

(D) Western blot analysis for the expression of Alix, CD63, CD81 and Cytochrome C proteins in HUVEC-derived exosomes (EXO), supernatant and total cell lysate (HUVEC). 50 μg total protein from cell lysate or EXOs was loaded. Cytochrome C was used as an organelle marker.

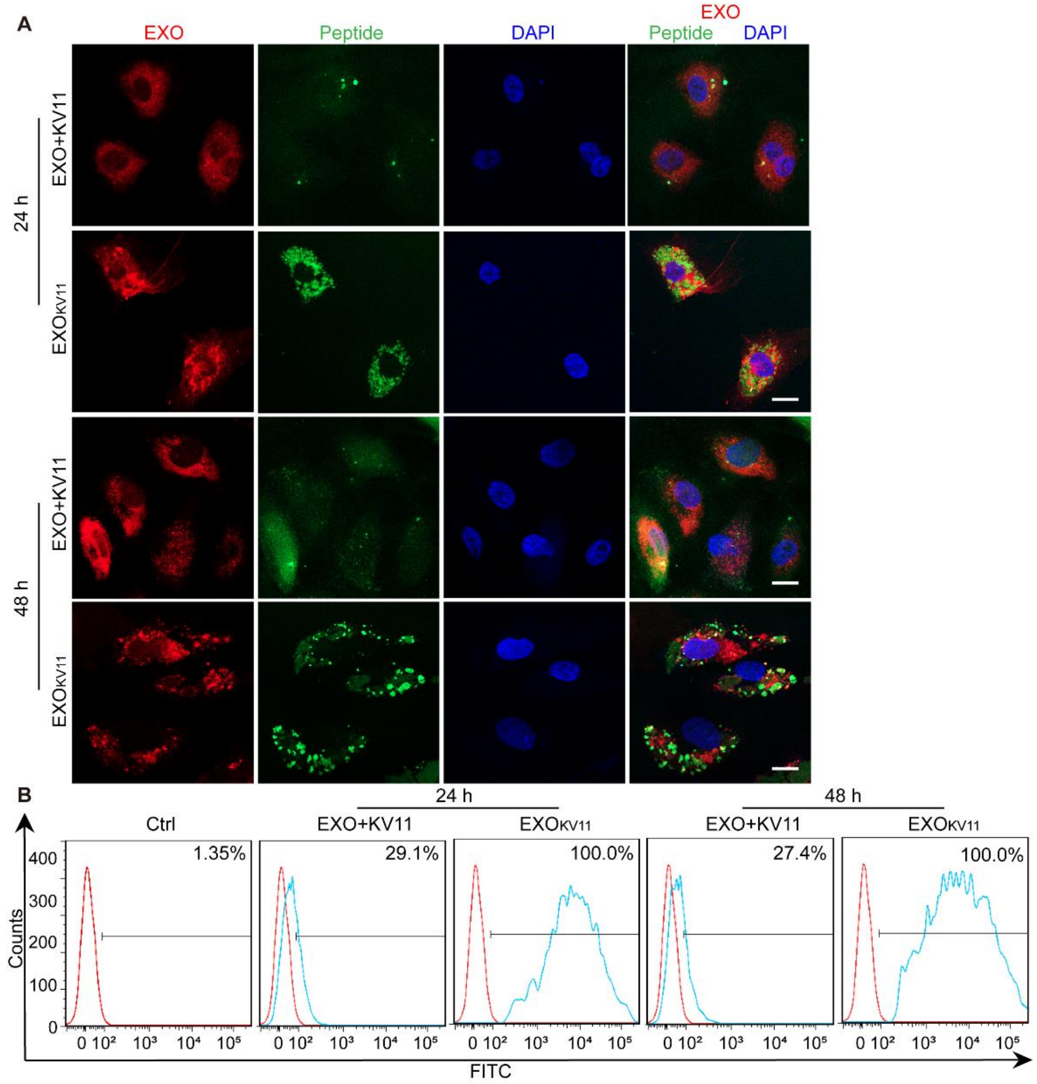

Figure S2. Cellular uptake of KV11 in HRMECs.

(A) DiR labled EXOs were incubated with FITC- labeled KV11 (EXO+KV11) or FITC-labeled KV11-CP05 (EXO_{KV11}). After incubation, each mixture was added to HRMECs in culture. Representative confocal images from at least three different experiments show the cellular uptake in HRMECs at 24 and 48 h.

(B) EXO+KV11 and EXO_{KV11} obtained as in (A) were added to HRMECs in culture. After 24 or 48 h, cells were trypsinized for flow cytometry to analyze the cellular uptake efficiency. The red line represents the no-treatment control (Ctrl). The percentage of FITC-positive events out of total events is indicated in each histogram.

Scale bars, 20 μm in (A)

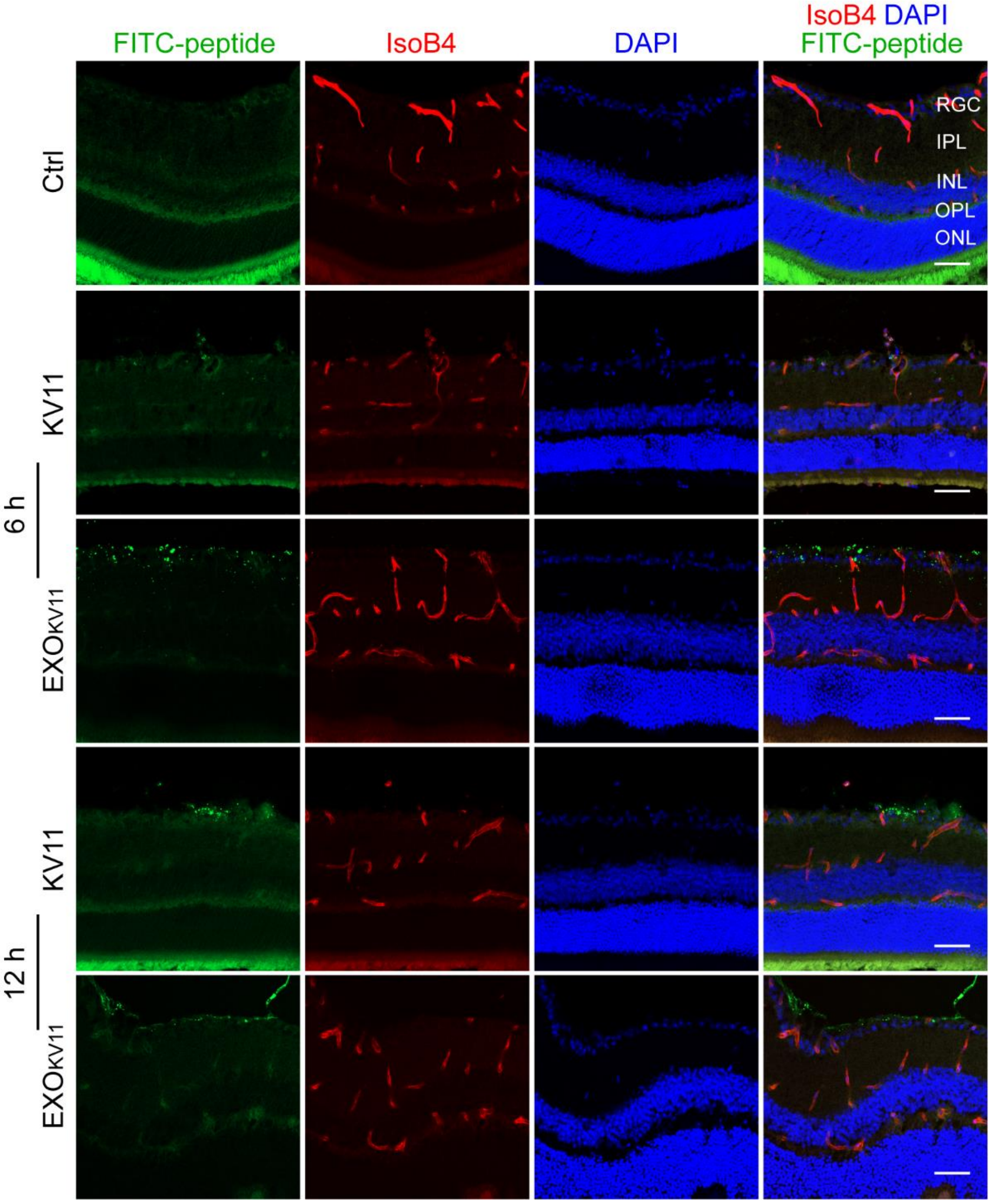

Figure S3. Ocular distribution of KV11 and EXO$_{KV11}$ after intravitreal injection.

KV11 or EXO$_{KV11}$ was injected intravitreally into CD-1 mice. Confocal images show the ocular distribution of KV11 and EXO$_{KV11}$ in cryosections of retinas harvested at the indicated time points (6 h or 12 h). IsoB4 was used to label blood vessels. Ctrl represents the uninjected group. For checking *in vivo* distribution, FITC-labeled peptides were used. Images are representative of at least three mice per condition. Scale bars, 20 μm.

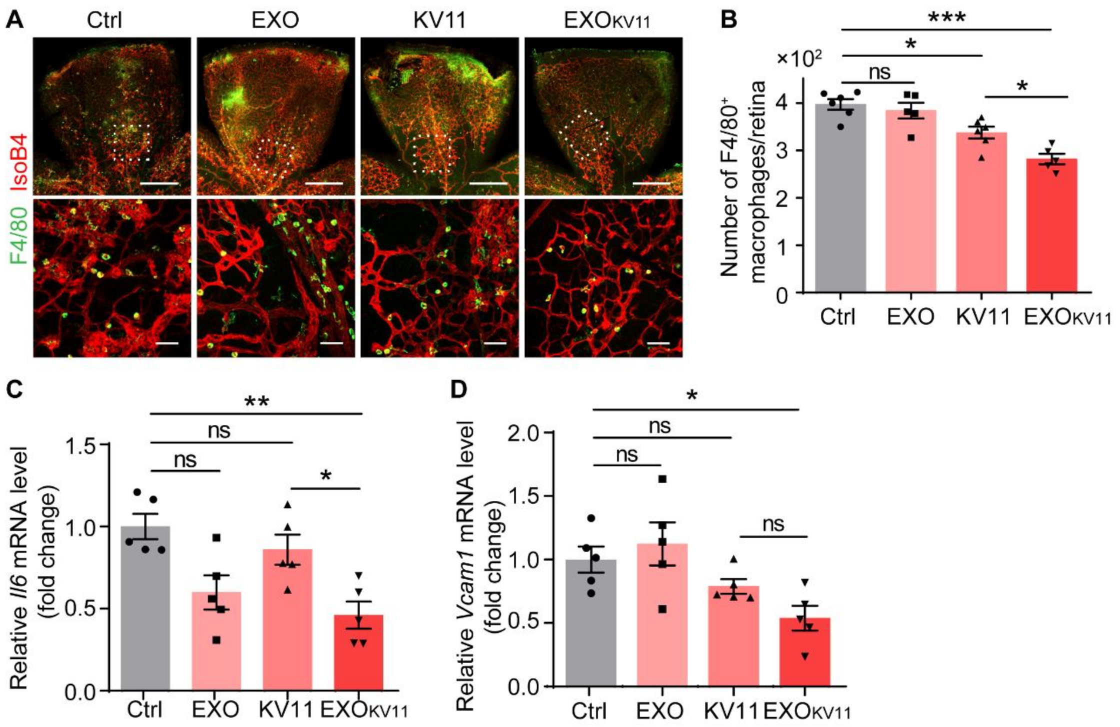

Figure S4. EXO$_{KV11}$ inhibits inflamation in the OIR model

(A and B) Representative confocal images (A) of F4/80$^+$ macrophage infiltration (green) and quantification (B) (n = 5 - 6 mice per condition).

(C and D) qPCR analysis of the expression of *Il6* and *Vcam*1 in retinas of saline vehicle (Ctrl)-, EXO-, KV11-, and EXO$_{KV11}$-treated pups at P17 (n = 5 mice per condition).

The data represent as mean ± SEM, *p < 0.05, **p < 0.01, ***p < 0.001, one-way ANOVA followed by Tukey's multiple comparisons test in (C), (E), (H), (I), (J).

Scale bars, 1 mm in (A); 500 µm (lower magnification) and 50 µm (insets) in (B) and (G); 50 µm in (D and F).

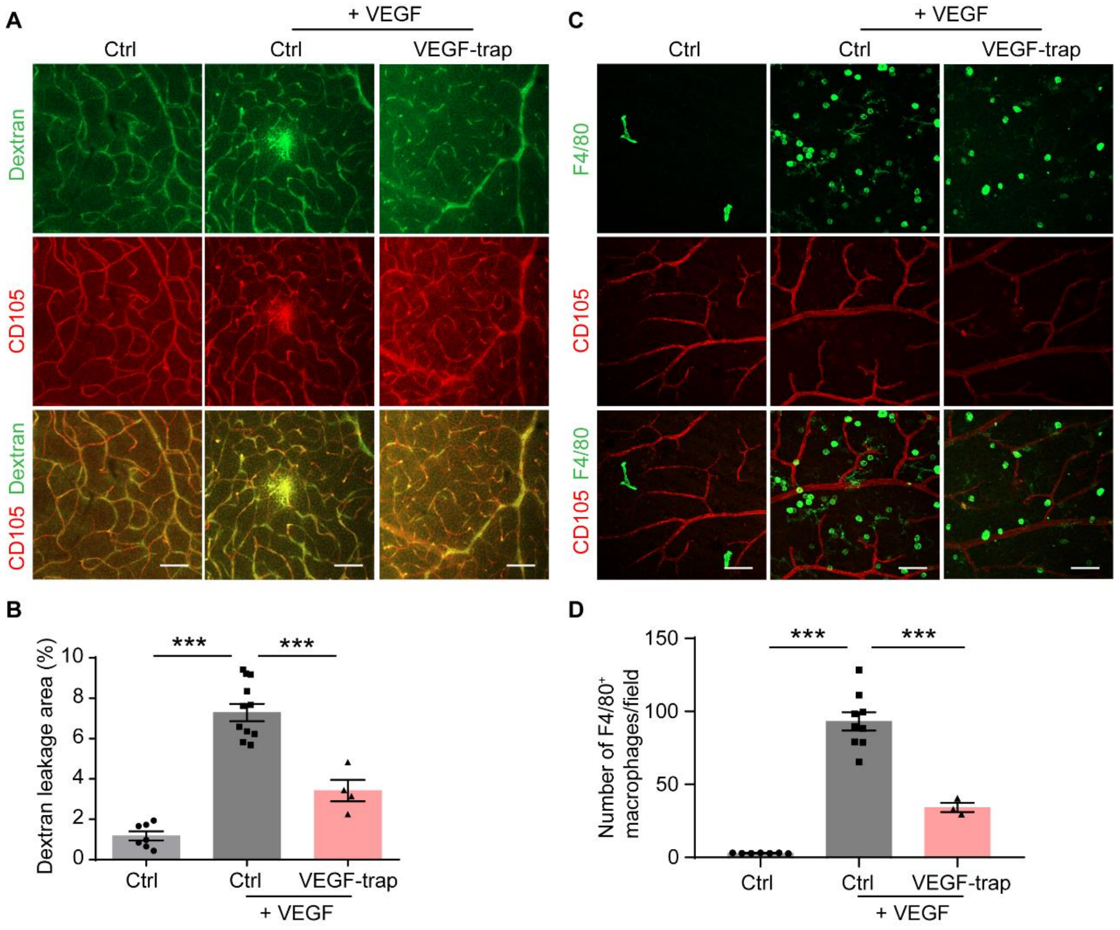

Figure S5. VEGF-trap suppresses VEGF-induced vascular leakage *in vivo*.

(A) VEGF-trap (15 μg; 1.5 μL of 10 mg/mL Conbercept) was intravitreally injected into adult mice. 100 ng VEGF was then intravitreally injected to induce vascular leakage in the retina as performed in Figure 5A. 24 h after VEGF administration, FITC-dextran was injected and the retinas were dissected. Representative images of flat-mounted retina showing extravasated FITC-dextran and $CD105^+$ vessels.

(B) Quantification of dextran leakage of (A) (n= 4 - 10 mice per condition).

(C) Representative images of retinas treated as in (A) immunostained for macrophage (F4/80) and blood vessels (CD105).

(D) Quantification of macrophage infiltration of (C) (n= 3 - 9 mice per condition).

The data represent as mean ± SEM. ***$p < 0.001$, one-way ANOVA followed by Tukey's multiple comparisons test in (B), (D).

Scale bars, 50 μm.

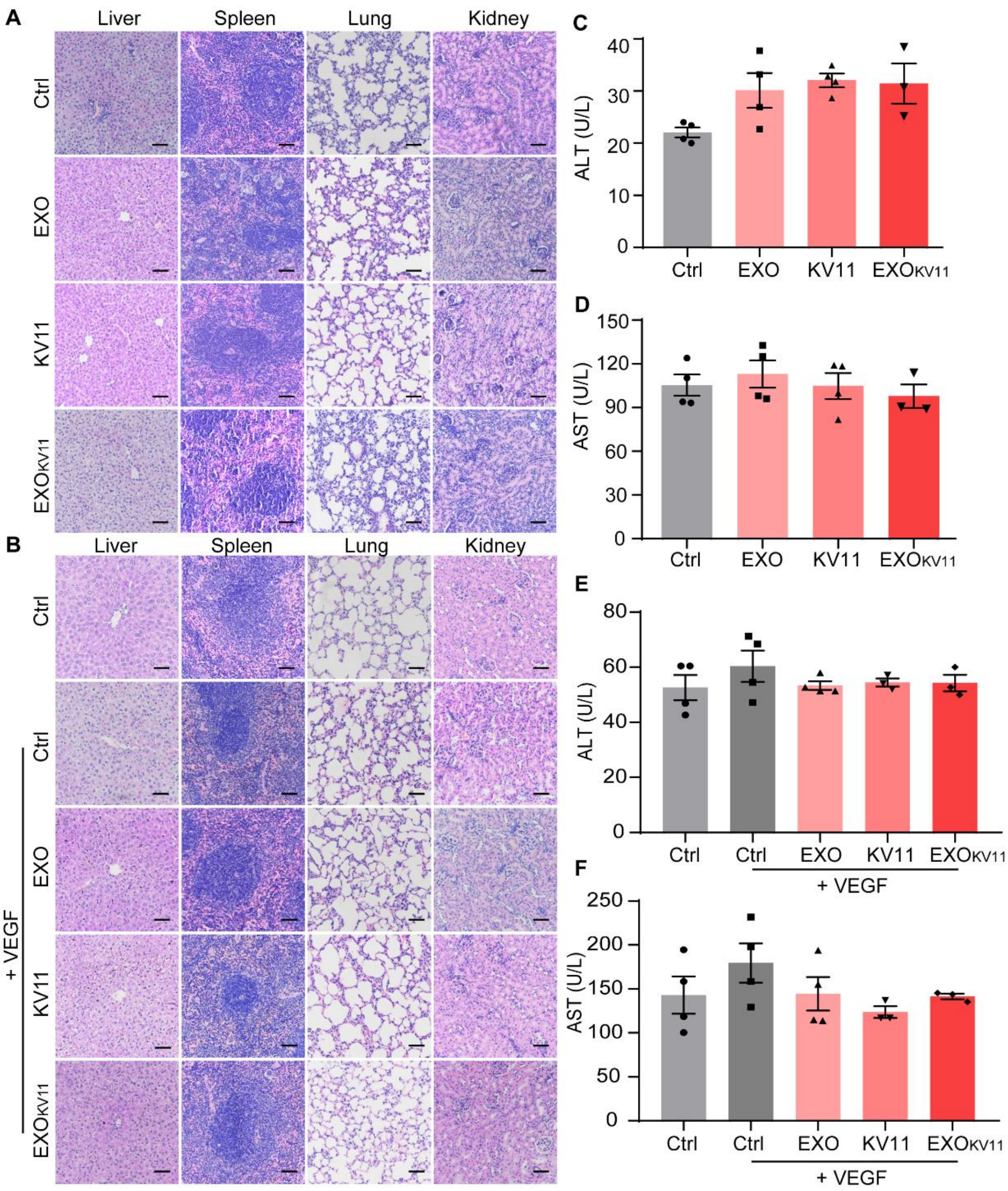

Figure S6. Assessment of organ toxicity in EXO_{KV11}-treated mice.

(A and B) Represetative images of hematoxylin and eosin staining of tissue sections from saline-treated controls (Ctrl), EXO-, KV11-, or EXO_{KV11}-treated mice in the OIR at P17 (A) and pre-treated VEGF-induced vascular leakage model at indicated endpoint (B).

(C, D, E and F) Measurement of serum alanine aminotransferase (ALT) and aspartate aminotransferase (AST) activity in mice treated as in (A and B)

The data represent as mean ± SEM, one-way ANOVA followed by Tukey's multiple comparisons test in (E), (F), (G), (H).

Scale bars, 50 μm.

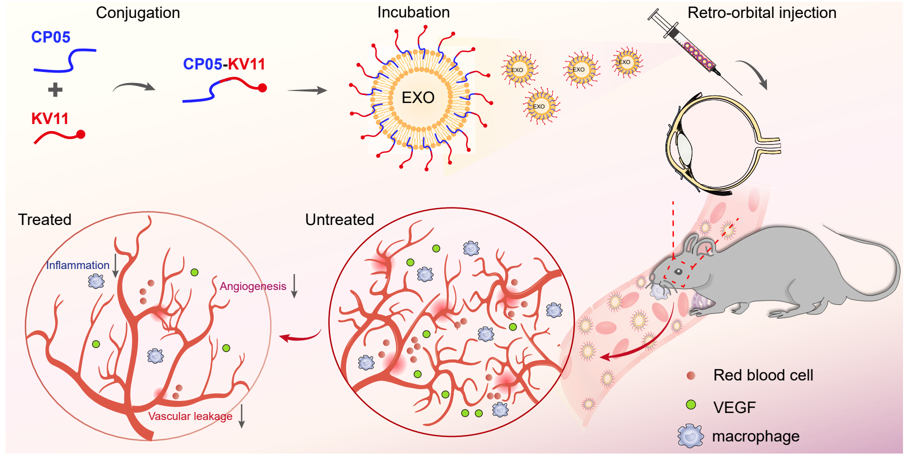

Conjugation
CP05
KV11
CP05-KV11
Incubation
EXO
Retro-orbital injection
Treated
Inflammation
Angiogenesis
Vascular leakage
Untreated
Red blood cell
VEGF
macrophage

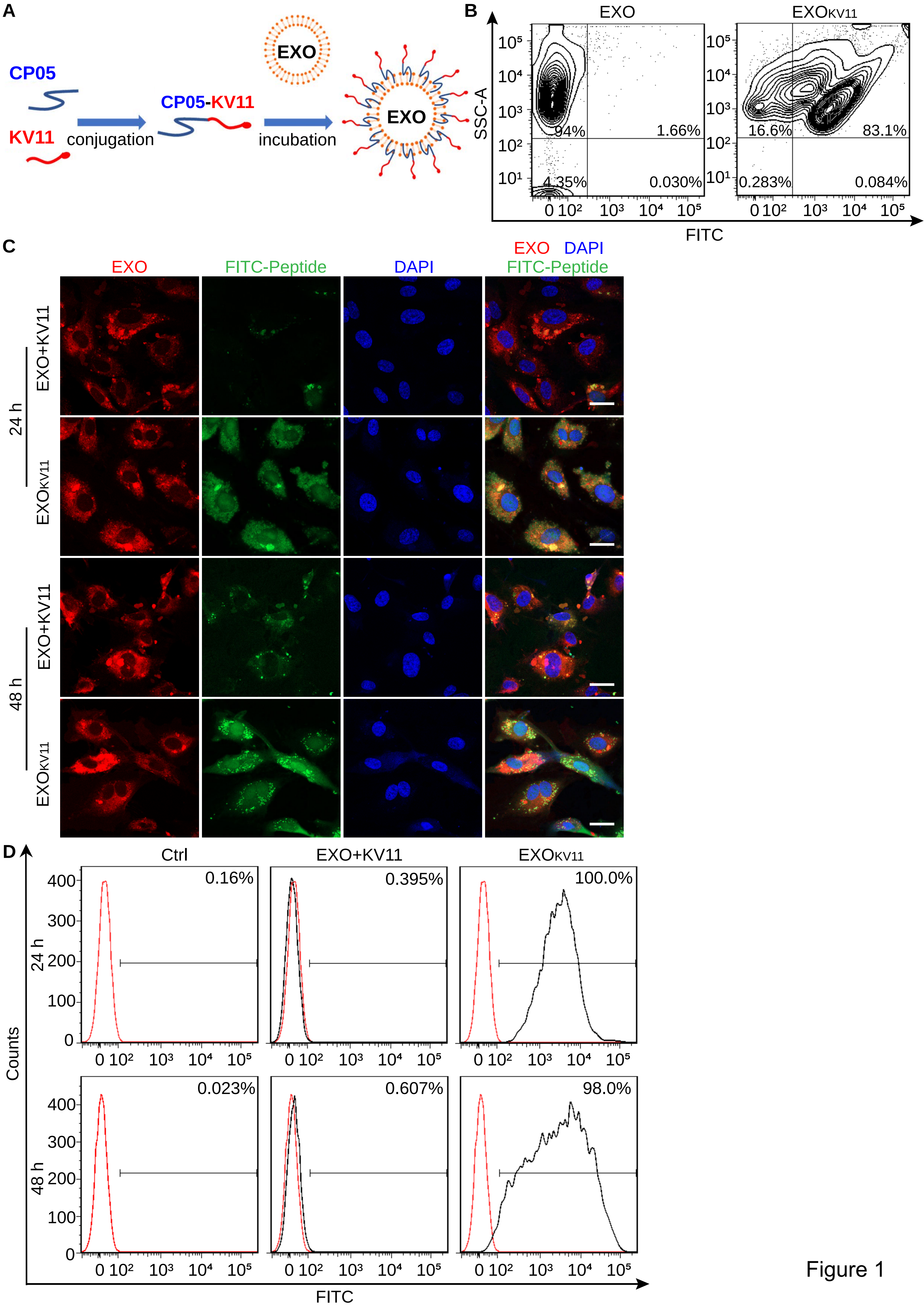
A
CP05
KV11
conjugation
CP05-KV11
incubation
EXO
B
EXO
EXOKV11
SSC-A
FITC
94%
1.66%
4.35%
0.030%
16.6%
83.1%
0.283%
0.084%
C
EXO
FITC-Peptide
DAPI
EXO DAPI FITC-Peptide
24 h
48 h
EXO+KV11
EXOKV11
D
Ctrl
EXO+KV11
EXOKV11
0.16%
0.395%
100.0%
0.023%
0.607%
98.0%
Counts
FITC

Figure 1

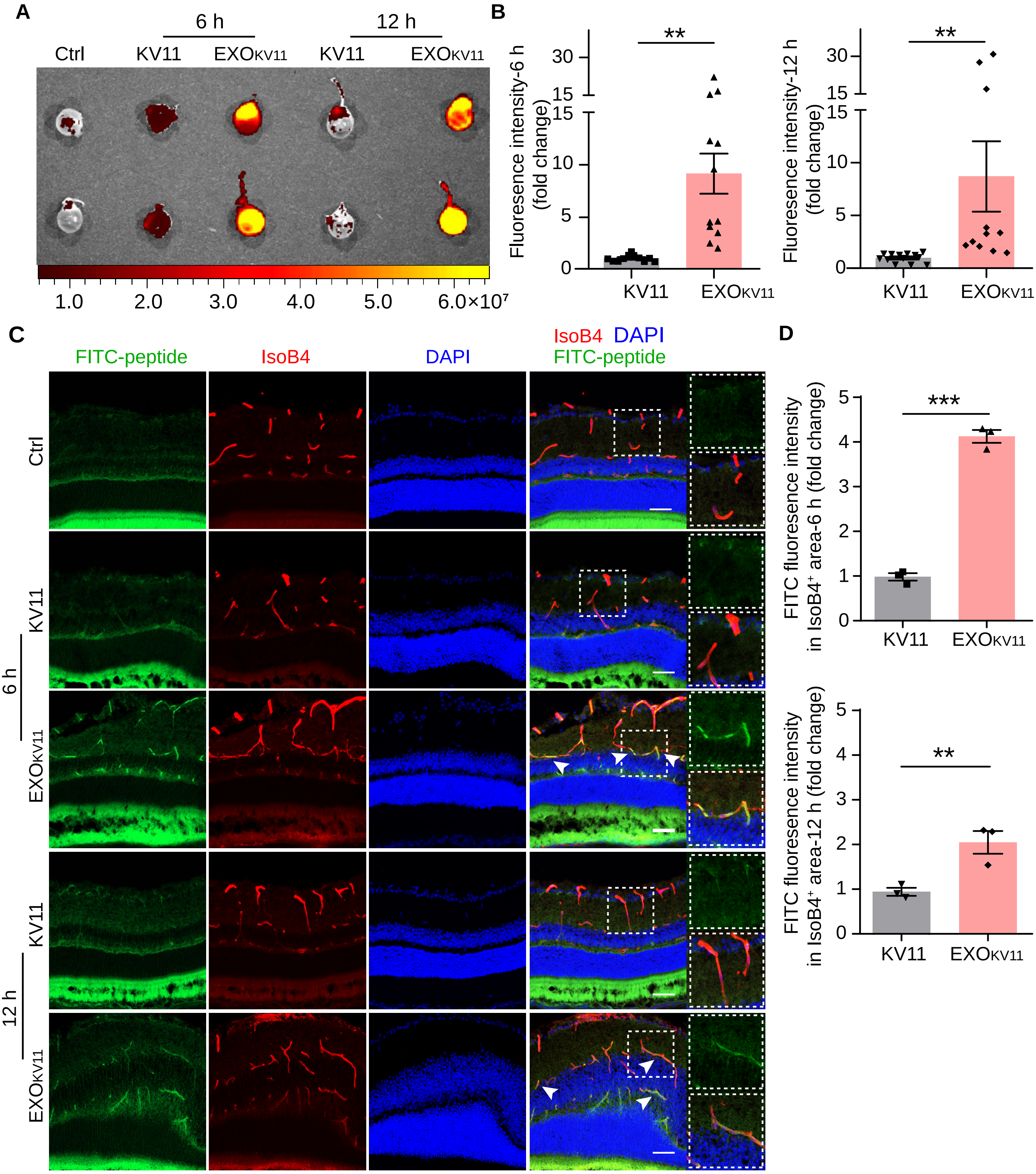
A
6 h
12 h
Ctrl
KV11
EXOKV11
KV11
EXOKV11
1.0
2.0
3.0
4.0
5.0
6.0×10⁷
B
Fluoresence intensity-6 h (fold change)
Fluoresence intensity-12 h (fold change)
**
**
KV11
EXOKV11
C
FITC-peptide
IsoB4
DAPI
IsoB4 DAPI FITC-peptide
Ctrl
KV11
EXOKV11
6 h
12 h
D
FITC fluoresence intensity in IsoB4+ area-6 h (fold change)
FITC fluoresence intensity in IsoB4+ area-12 h (fold change)
***
**

Figure 2

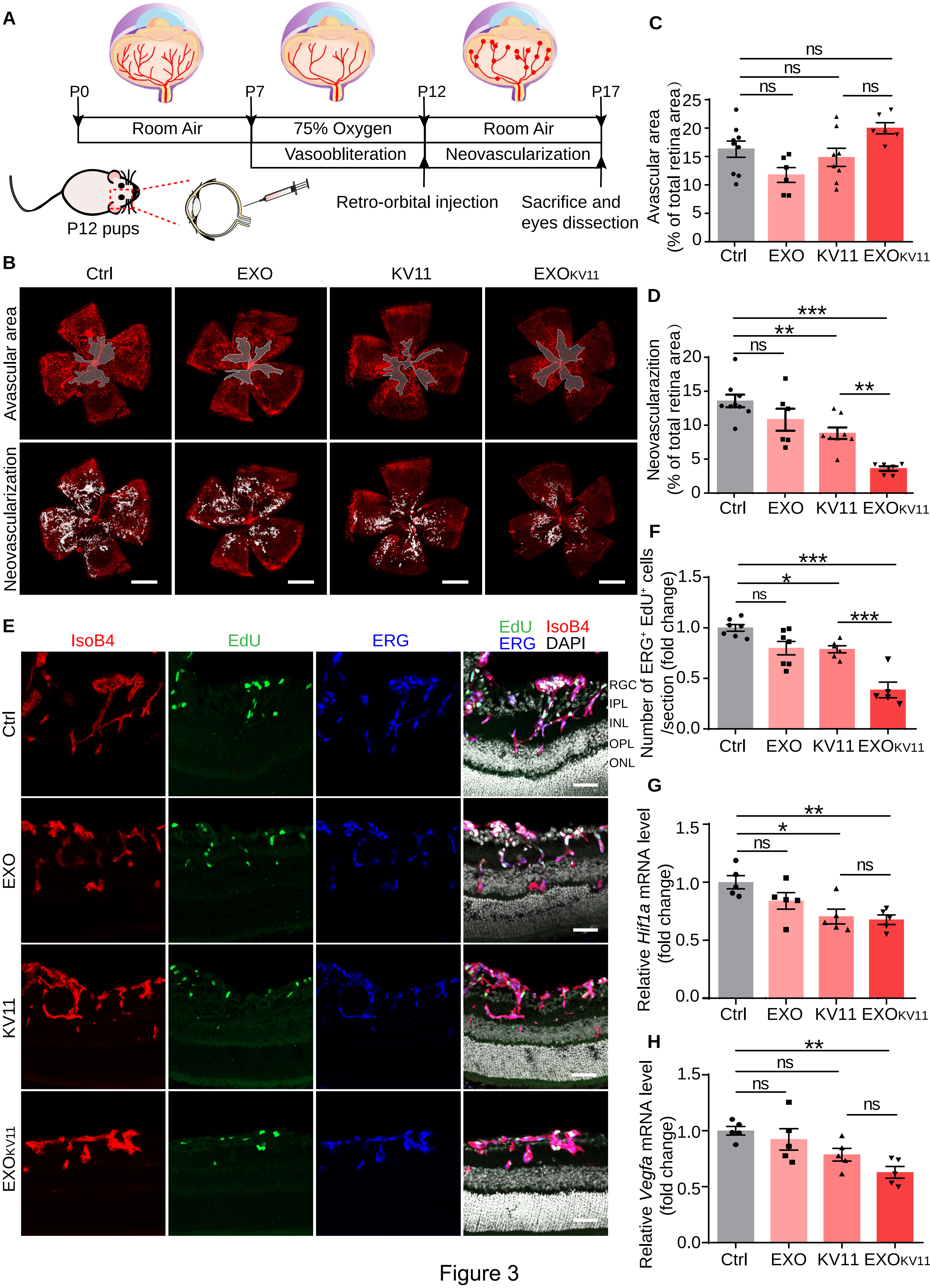
A
P0
P7
P12
P17
Room Air
75% Oxygen
Room Air
Vasoobliteration
Neovascularization
P12 pups
Retro-orbital injection
Sacrifice and eyes dissection
B
Ctrl
EXO
KV11
EXOKV11
Avascular area
Neovascularization
C
Avascular area (% of total retina area)
D
Neovascularazition (% of total retina area)
E
IsoB4
EdU
ERG
EdU IsoB4 ERG DAPI
RGC
IPL
INL
OPL
ONL
F
Number of ERG+ EdU+ cells /section (fold change)
G
Relative Hif1a mRNA level (fold change)
H
Relative Vegfa mRNA level (fold change)
ns
*
**
***

Figure 3

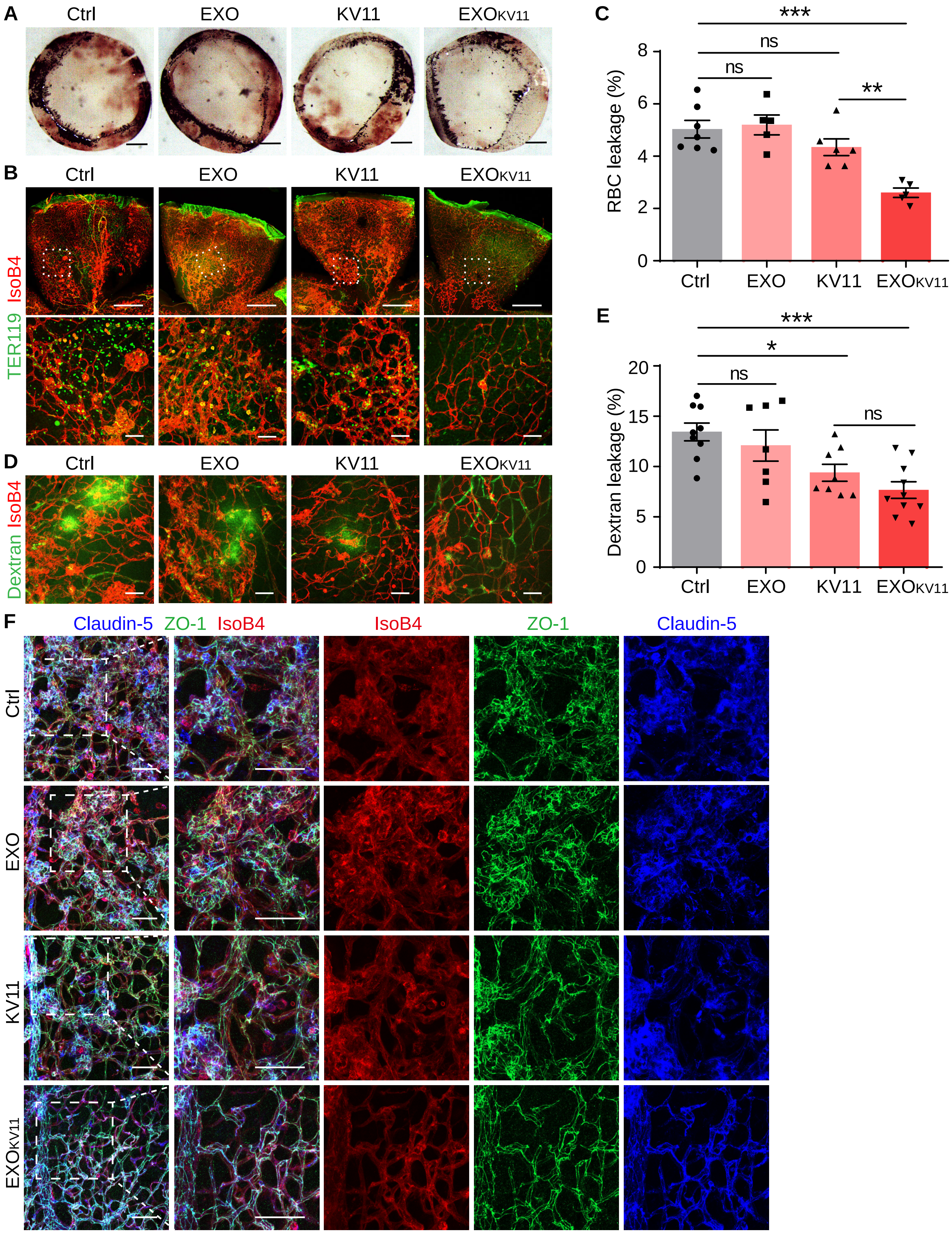
A
Ctrl
EXO
KV11
EXOKV11
B
Ctrl
EXO
KV11
EXOKV11
TER119 IsoB4
C
RBC leakage (%)
0
2
4
6
8
***
ns
ns
**
Ctrl
EXO
KV11
EXOKV11
D
Ctrl
EXO
KV11
EXOKV11
Dextran IsoB4
E
Dextran leakage (%)
0
5
10
15
20
***
*
ns
ns
Ctrl
EXO
KV11
EXOKV11
F
Claudin-5 ZO-1 IsoB4
IsoB4
ZO-1
Claudin-5
Ctrl
EXO
KV11
EXOKV11

Figure 4

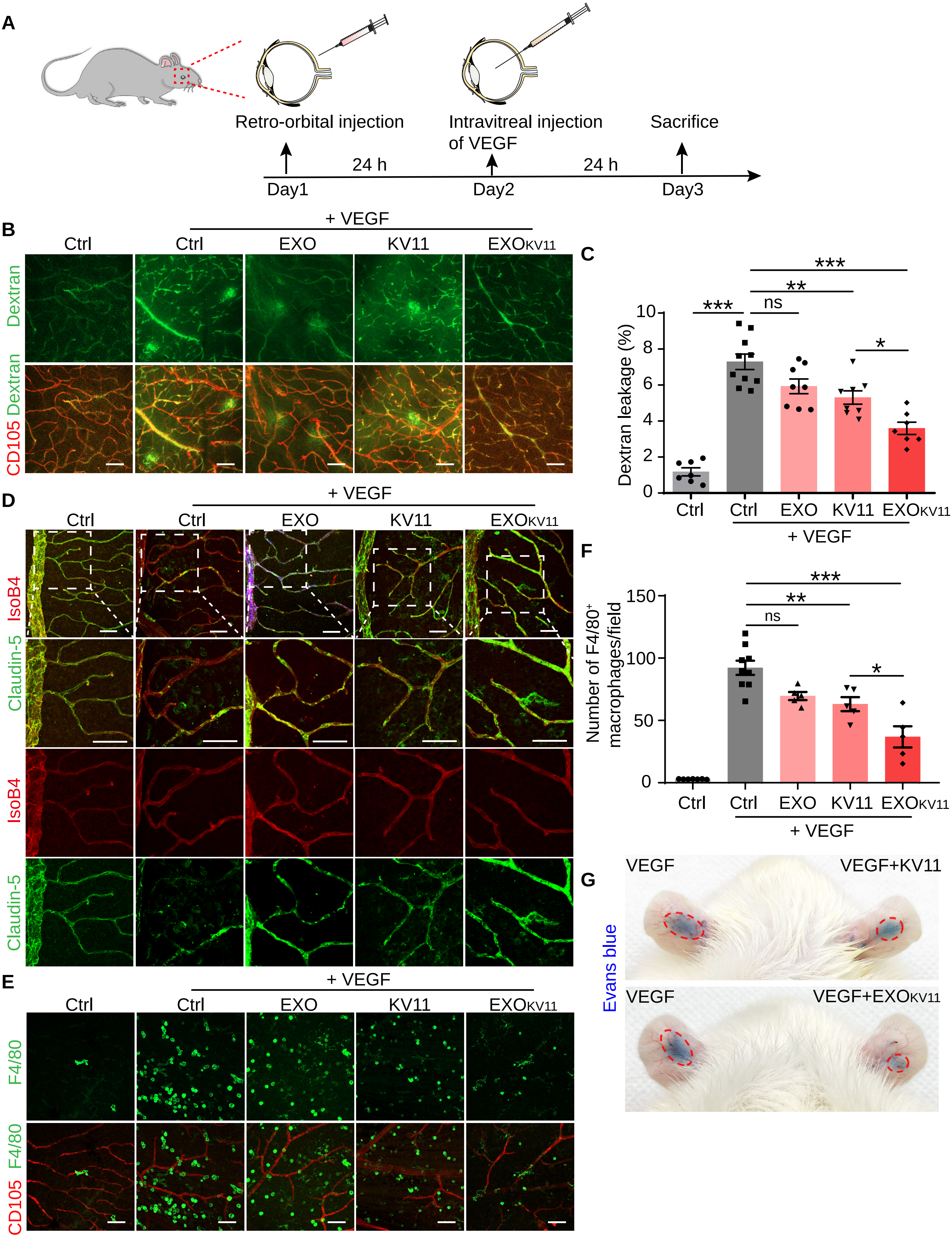
A
Retro-orbital injection
Intravitreal injection of VEGF
Sacrifice
24 h
24 h
Day1
Day2
Day3
B
+ VEGF
Ctrl
Ctrl
EXO
KV11
EXOKV11
Dextran
CD105 Dextran
C
Dextran leakage (%)
***
ns
**
***
*
Ctrl
Ctrl
EXO
KV11
EXOKV11
+ VEGF
D
+ VEGF
Ctrl
Ctrl
EXO
KV11
EXOKV11
IsoB4
Claudin-5
IsoB4
Claudin-5
F
Number of F4/80+ macrophages/field
ns
**
***
*
Ctrl
Ctrl
EXO
KV11
EXOKV11
+ VEGF
G
Evans blue
VEGF
VEGF+KV11
VEGF
VEGF+EXOKV11
E
+ VEGF
Ctrl
Ctrl
EXO
KV11
EXOKV11
F4/80
CD105 F4/80

Figure 5

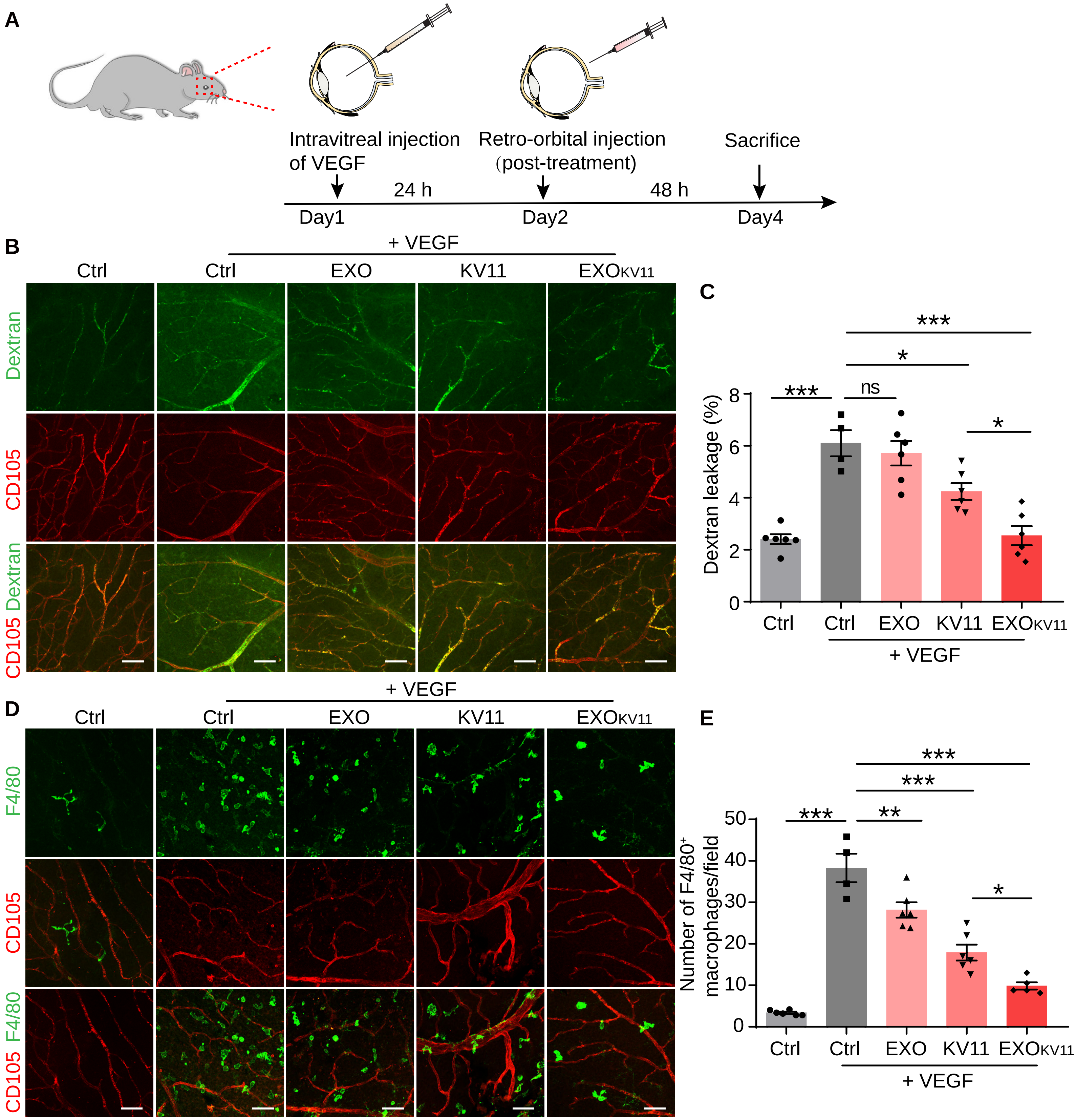
A
Intravitreal injection of VEGF
Retro-orbital injection (post-treatment)
Sacrifice
24 h
48 h
Day1
Day2
Day4
B
+ VEGF
Ctrl
Ctrl
EXO
KV11
EXOKV11
Dextran
CD105
CD105 Dextran
C
Dextran leakage (%)
***
ns
*
***
*
Ctrl
Ctrl
EXO
KV11
EXOKV11
+ VEGF
D
+ VEGF
Ctrl
Ctrl
EXO
KV11
EXOKV11
F4/80
CD105
CD105 F4/80
E
Number of F4/80+ macrophages/field
***
**
***
***
*
Ctrl
Ctrl
EXO
KV11
EXOKV11
+ VEGF

Figure 6

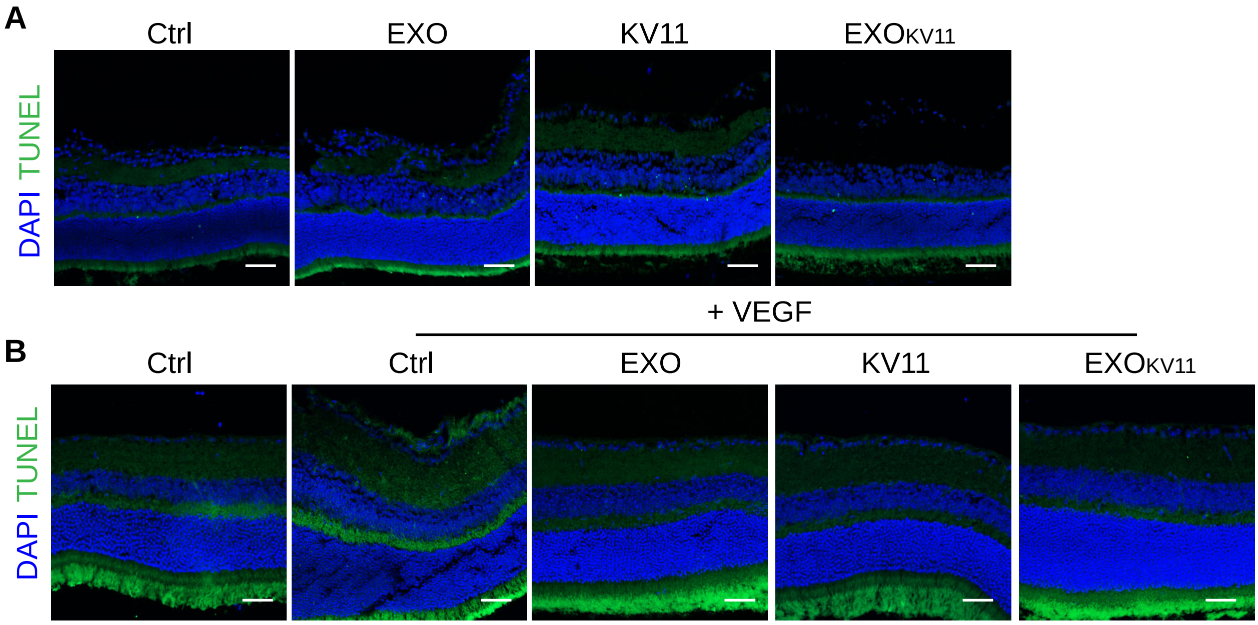
A
Ctrl
EXO
KV11
EXOKV11
DAPI TUNEL
+ VEGF
B
Ctrl
Ctrl
EXO
KV11
EXOKV11
DAPI TUNEL

Figure 7

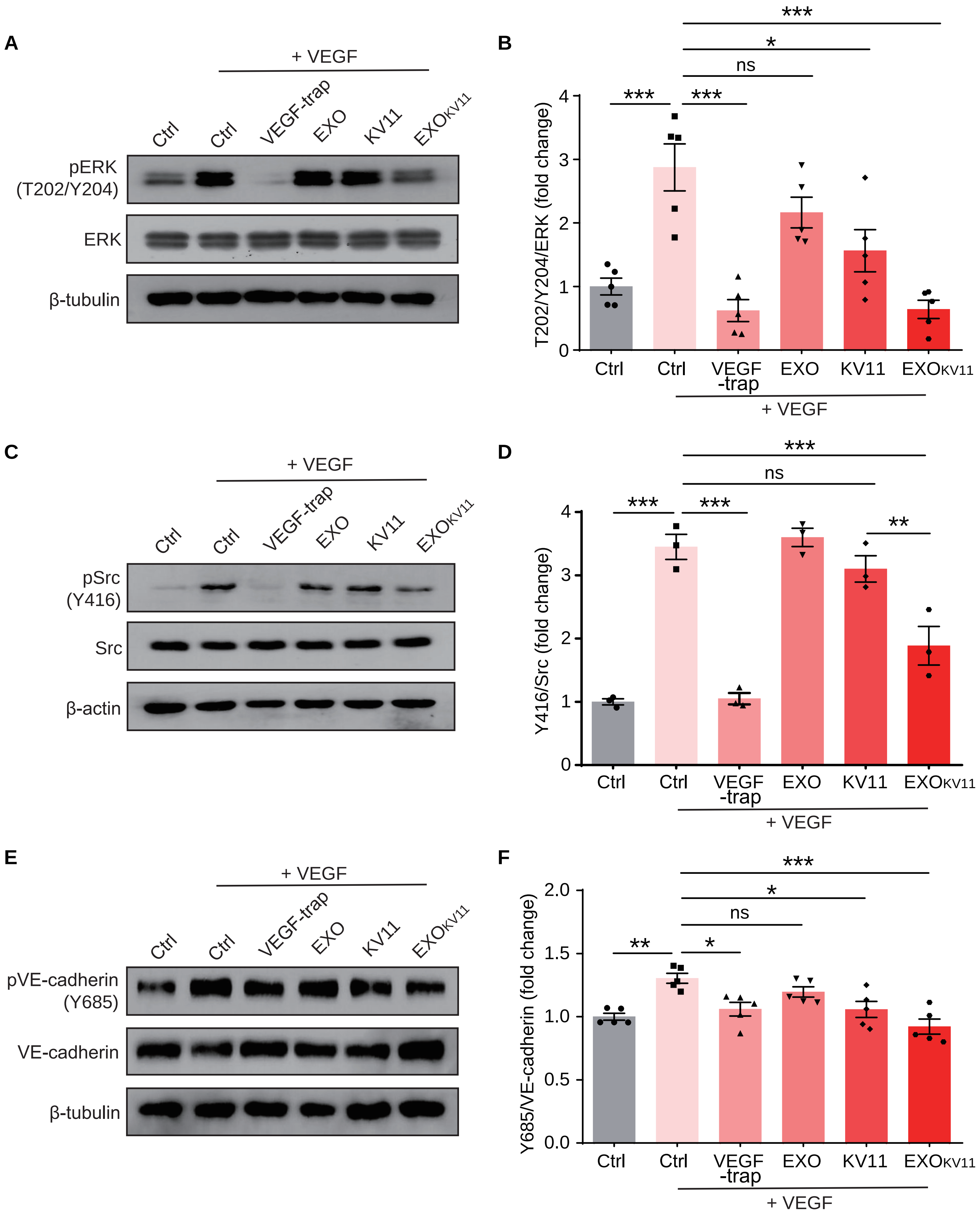

A
+ VEGF
Ctrl
Ctrl
VEGF-trap
EXO
KV11
EXOKV11
pERK (T202/Y204)
ERK
β-tubulin
B
T202/Y204/ERK (fold change)
***
*
ns
***
***
Ctrl
Ctrl
VEGF -trap
EXO
KV11
EXOKV11
+ VEGF
C
+ VEGF
pSrc (Y416)
Src
β-actin
D
Y416/Src (fold change)
***
ns
***
***
**
E
+ VEGF
pVE-cadherin (Y685)
VE-cadherin
β-tubulin
F
Y685/VE-cadherin (fold change)
***
*
ns
**
*

Figure 8

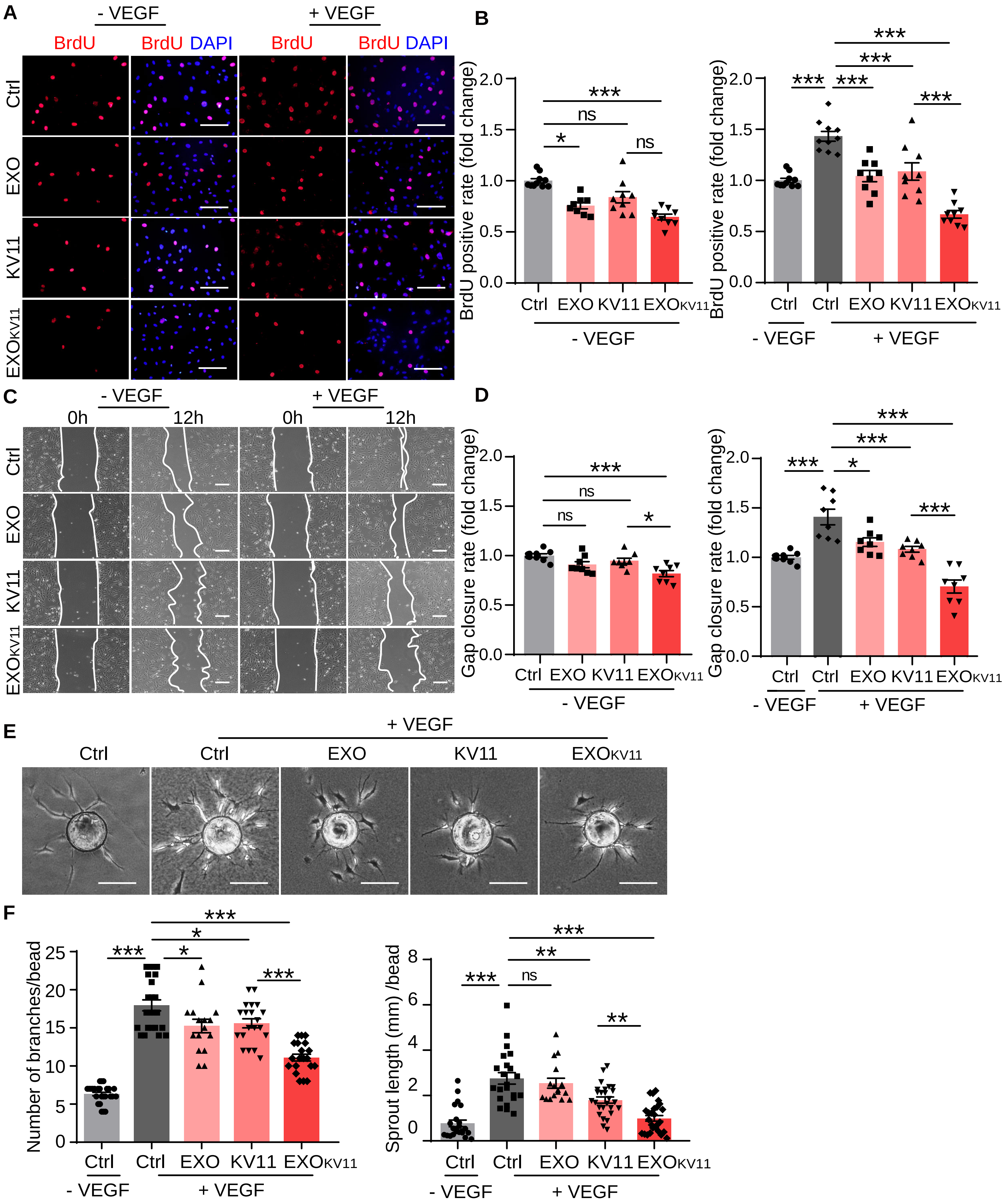
A
- VEGF
+ VEGF
BrdU
BrdU DAPI
Ctrl
EXO
KV11
EXOKV11
B
BrdU positive rate (fold change)
Ctrl EXO KV11 EXOKV11
- VEGF
+ VEGF
***
ns
*
C
0h
12h
D
Gap closure rate (fold change)
E
F
Number of branches/bead
Sprout length (mm) /bead
**

Figure 9

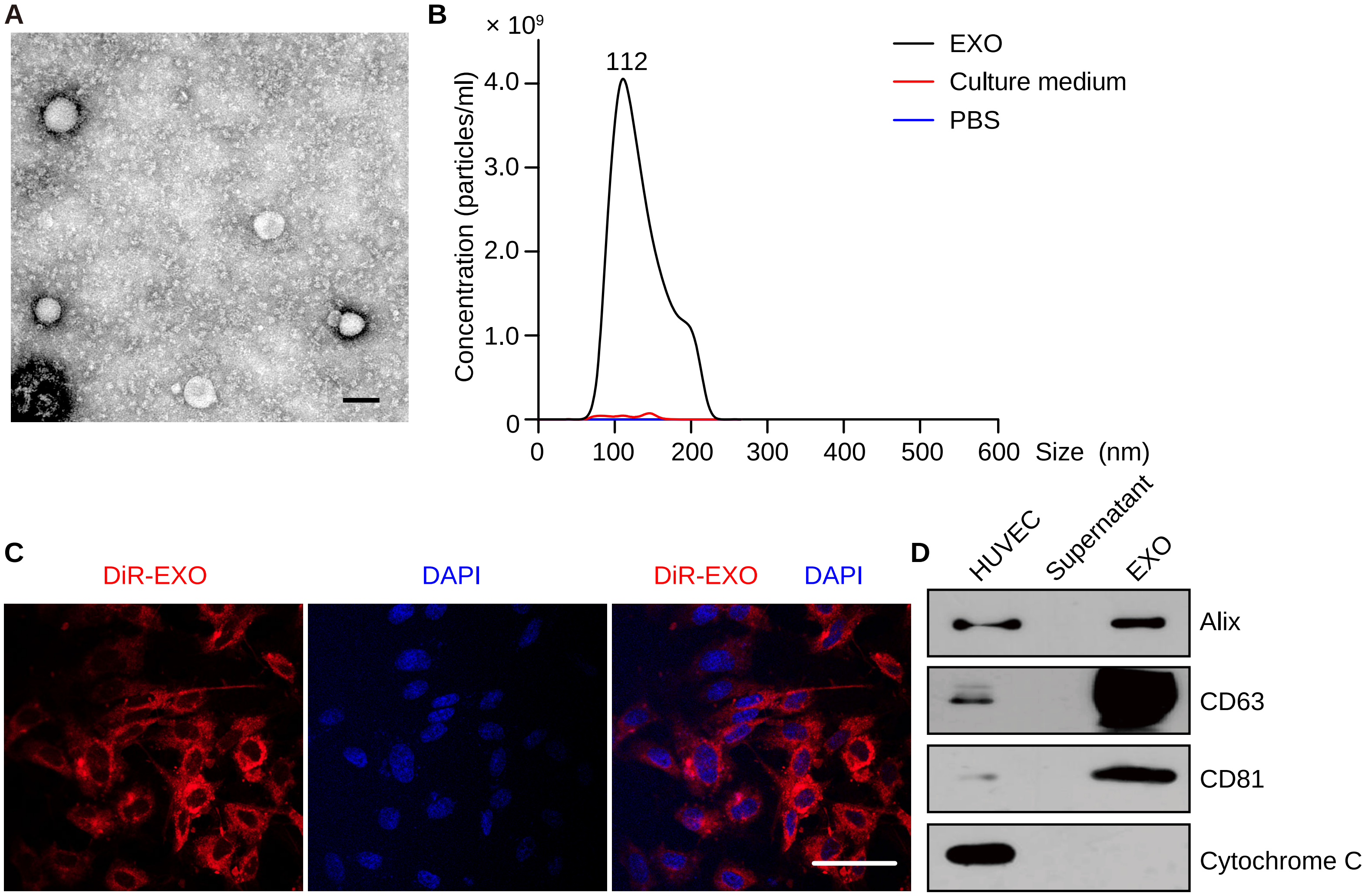
A
B
× 10⁹
112
4.0
3.0
2.0
1.0
0
Concentration (particles/ml)
0
100
200
300
400
500
600
Size (nm)
EXO
Culture medium
PBS
C
DiR-EXO
DAPI
DiR-EXO
DAPI
D
HUVEC
Supernatant
EXO
Alix
CD63
CD81
Cytochrome C

Figure S1

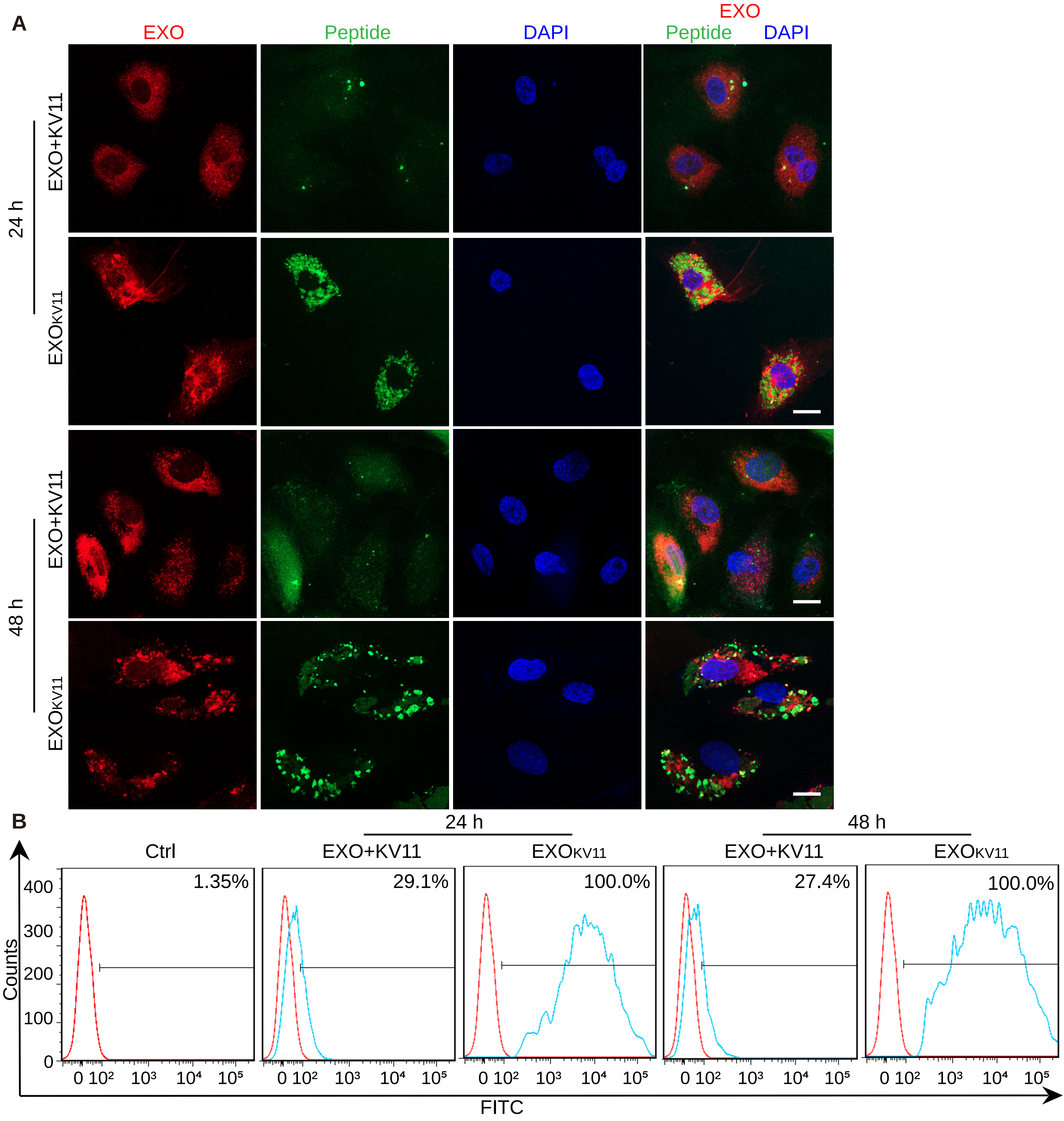
A
EXO
Peptide
DAPI
EXO
Peptide DAPI
24 h
EXO+KV11
EXOKV11
48 h
EXO+KV11
EXOKV11
B
24 h
48 h
Ctrl
EXO+KV11
EXOKV11
EXO+KV11
EXOKV11
1.35%
29.1%
100.0%
27.4%
100.0%
Counts
400
300
200
100
0
0 10²
10³
10⁴
10⁵
FITC

Figure S2

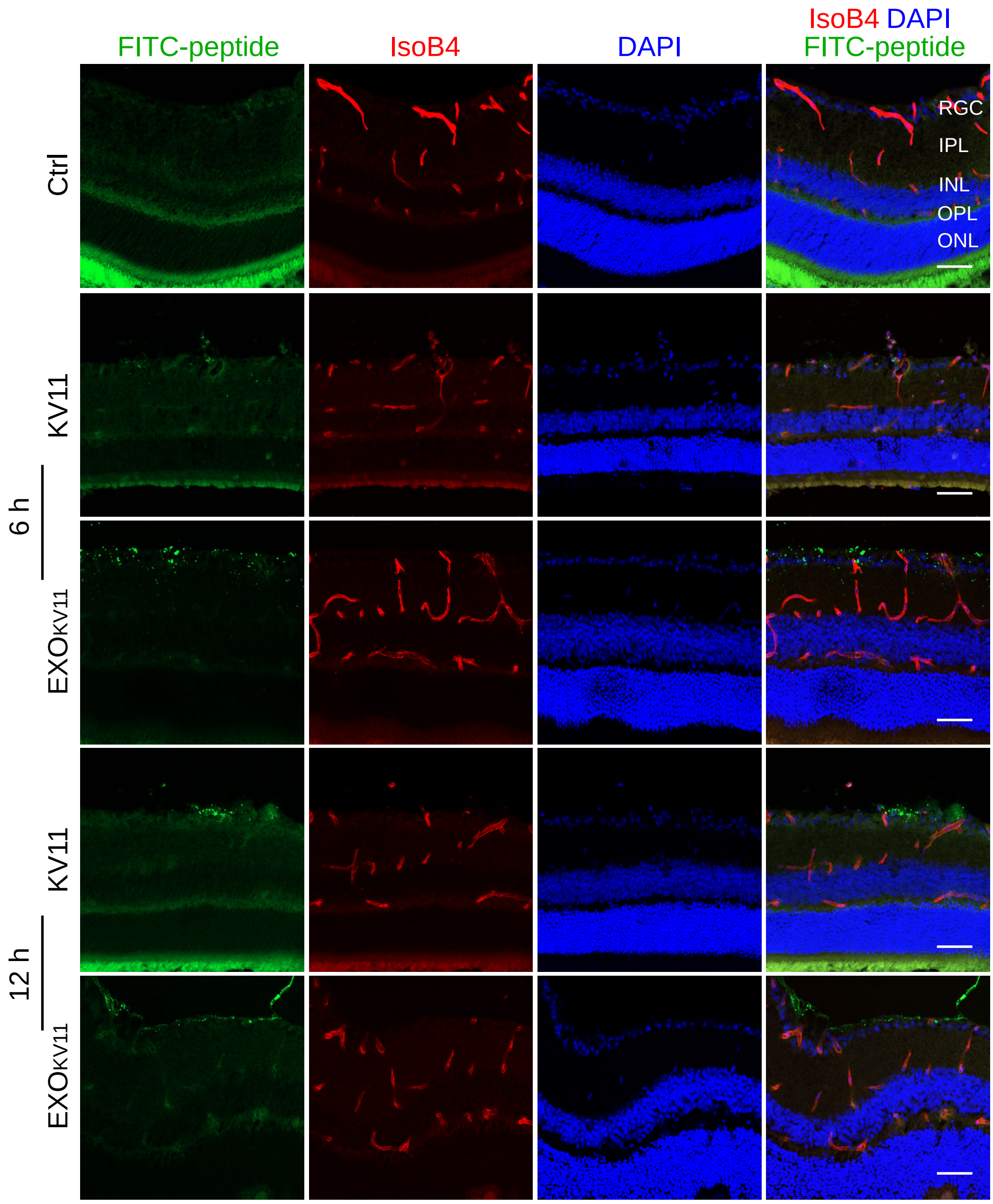
FITC-peptide
IsoB4
DAPI
IsoB4 DAPI
FITC-peptide
Ctrl
RGC
IPL
INL
OPL
ONL
KV11
6 h
EXOKV11
KV11
12 h
EXOKV11

Figure S3

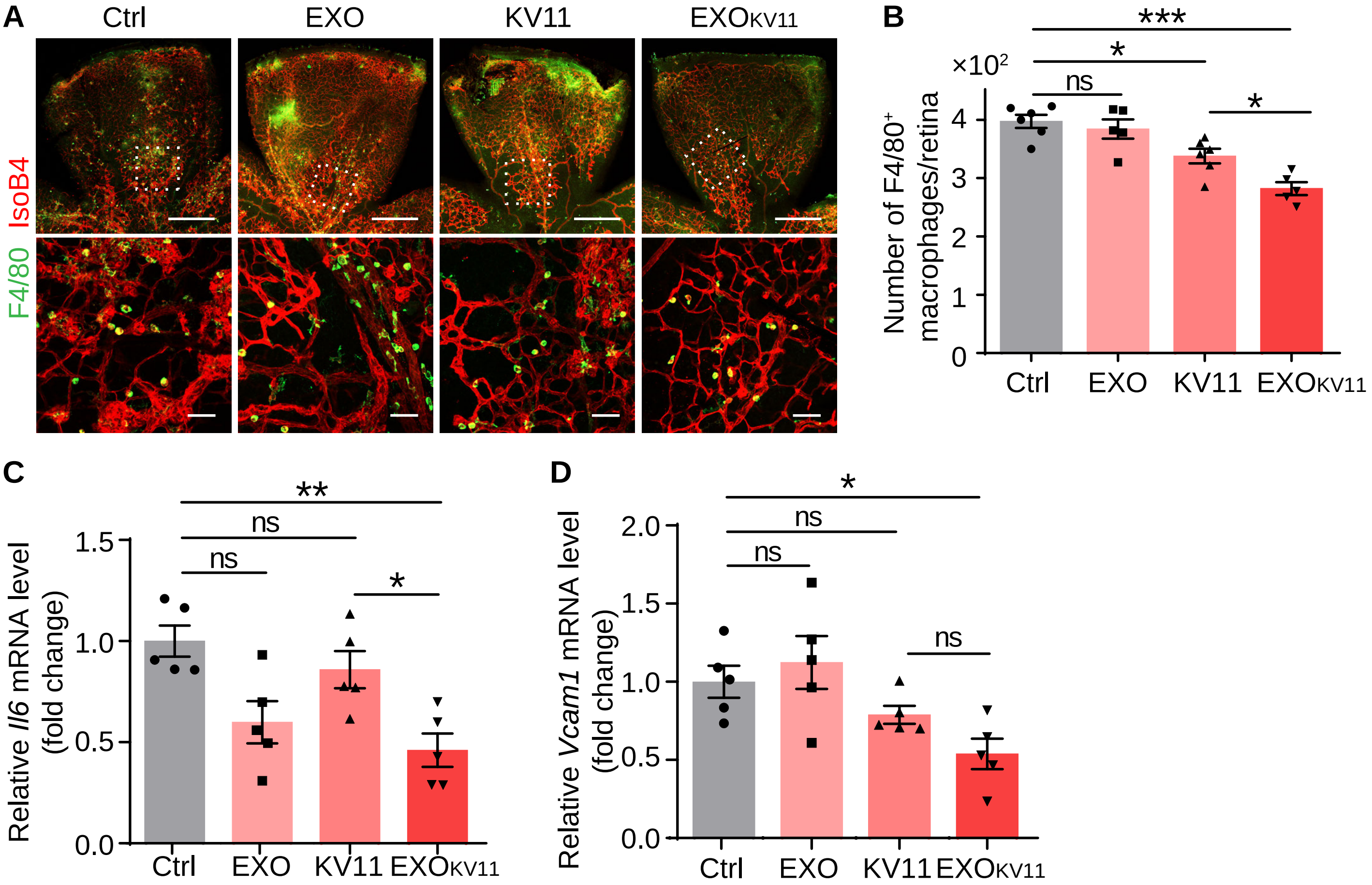

A
Ctrl
EXO
KV11
EXOKV11
IsoB4
F4/80
B
×10²
Number of F4/80⁺ macrophages/retina
***
*
ns
*
0
1
2
3
4
Ctrl
EXO
KV11
EXOKV11
C
Relative Il6 mRNA level (fold change)
**
ns
ns
*
0.0
0.5
1.0
1.5
Ctrl
EXO
KV11
EXOKV11
D
Relative Vcam1 mRNA level (fold change)
*
ns
ns
ns
0.0
0.5
1.0
1.5
2.0
Ctrl
EXO
KV11
EXOKV11

Figure S4

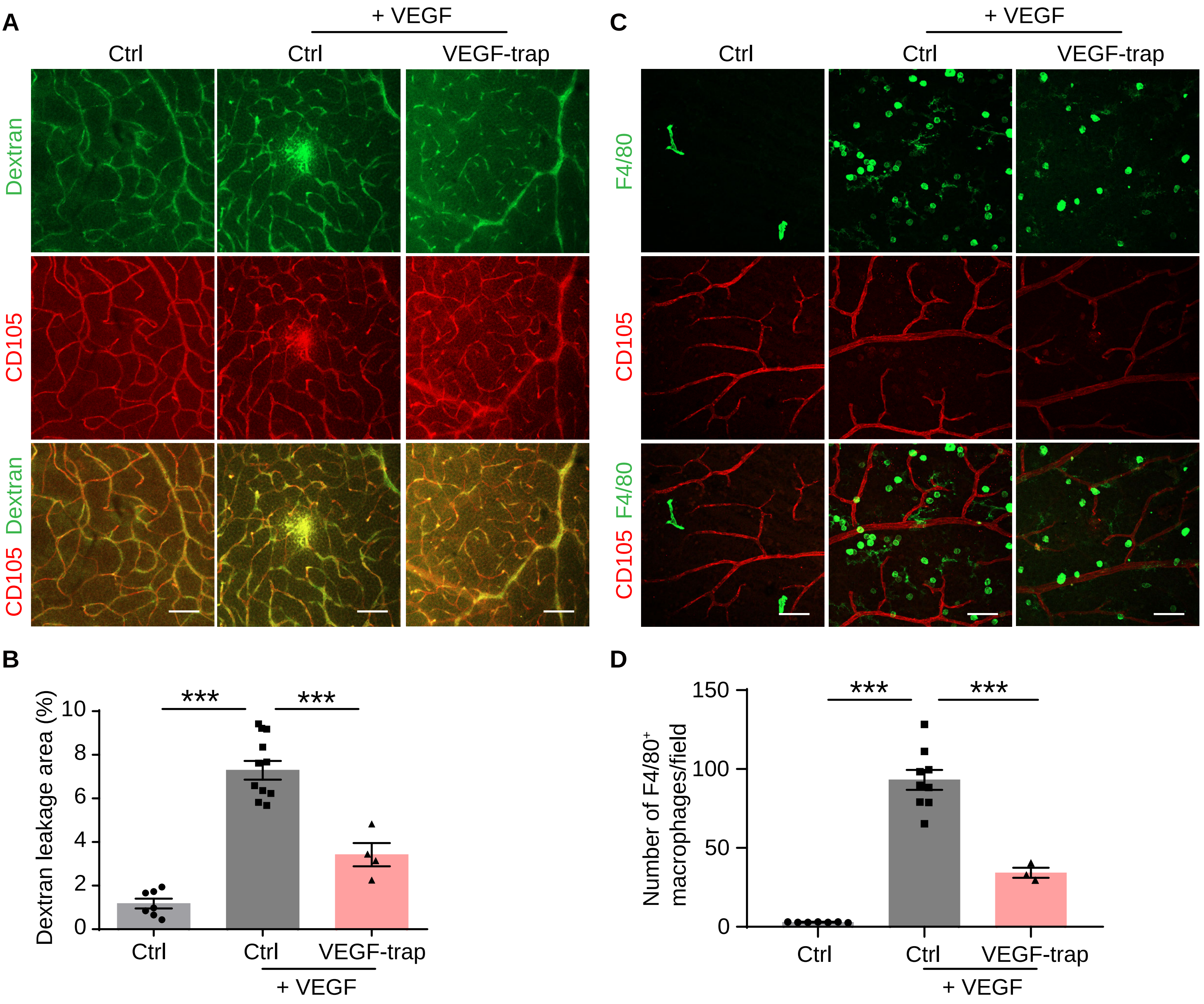
A
+ VEGF
Ctrl
Ctrl
VEGF-trap
Dextran
CD105
CD105 Dextran
B
Dextran leakage area (%)
10
8
6
4
2
0
***
***
Ctrl
Ctrl
VEGF-trap
+ VEGF
C
+ VEGF
Ctrl
Ctrl
VEGF-trap
F4/80
CD105
CD105 F4/80
D
Number of F4/80+ macrophages/field
150
100
50
0
***
***
Ctrl
Ctrl
VEGF-trap
+ VEGF

Figure S5

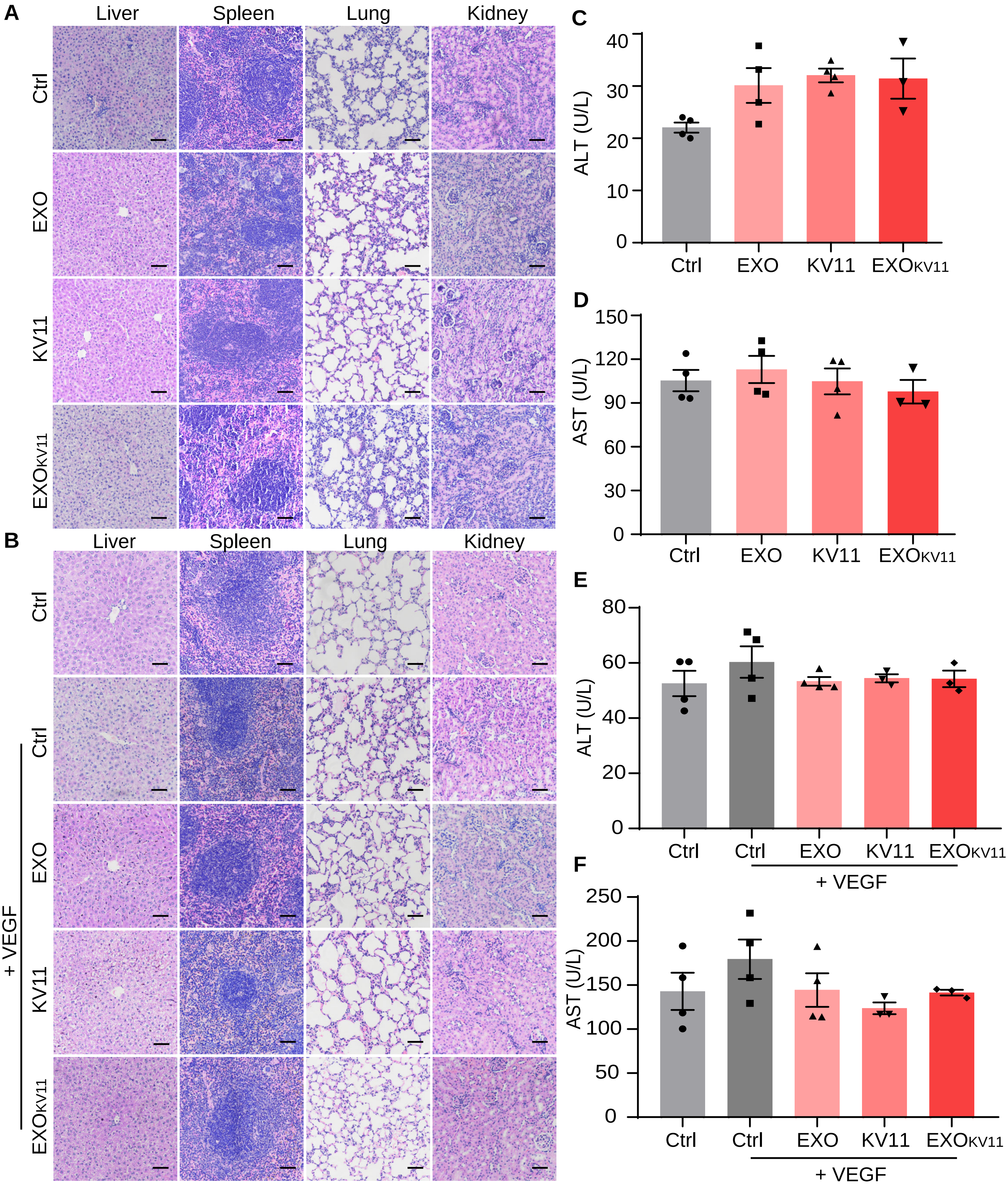
A
Liver
Spleen
Lung
Kidney
Ctrl
EXO
KV11
EXOKV11
B
Liver
Spleen
Lung
Kidney
Ctrl
Ctrl
EXO
KV11
EXOKV11
+ VEGF
C
ALT (U/L)
40
30
20
10
0
Ctrl
EXO
KV11
EXOKV11
D
AST (U/L)
150
120
90
60
30
0
Ctrl
EXO
KV11
EXOKV11
E
ALT (U/L)
80
60
40
20
0
Ctrl
Ctrl
EXO
KV11
EXOKV11
+ VEGF
F
AST (U/L)
250
200
150
100
50
0
Ctrl
Ctrl
EXO
KV11
EXOKV11
+ VEGF

Figure S6
